# Archaeological landscape, settlement dynamics, and sociopolitical organization in the Chactún area of the central Maya Lowlands

**DOI:** 10.1371/journal.pone.0262921

**Published:** 2022-01-21

**Authors:** Ivan Šprajc, Aleš Marsetič, Jasmina Štajdohar, Sara Dzul Góngora, Joseph W. Ball, Octavio Esparza Olguín, Žiga Kokalj

**Affiliations:** 1 Institute of Anthropological and Spatial Studies, Research Center of the Slovenian Academy of Sciences and Arts (ZRC SAZU), Ljubljana, Slovenia; 2 Centro INAH Yucatán, Instituto Nacional de Antropología e Historia, Mérida, Yuc., Mexico; 3 Department of Anthropology, San Diego State University, San Diego, CA, United States of America; 4 Centro de Estudios Mayas, Instituto de Investigaciones Filológicas, Universidad Nacional Autónoma de México, Ciudad de México, Mexico; New York State Museum, UNITED STATES

## Abstract

Until recently, an extensive area in the central lowlands of the Yucatán peninsula was completely unexplored archaeologically. In 2013 and 2014, during initial surveys in the northern part of the uninhabited Calakmul Biosphere Reserve in eastern Campeche, Mexico, we located Chactún, Tamchén and Lagunita, three major Maya centers with some unexpected characteristics. Lidar data, acquired in 2016 for a larger area of 240 km^2^, revealed a thoroughly modified and undisturbed archaeological landscape with a remarkably large number of residential clusters and widespread modifications related to water management and agriculture. Substantial additional information was obtained through field surveys and test excavations in 2017 and 2018. While hydraulic and agricultural features and their potential for solving various archaeologically relevant questions were discussed in a previous publication, here we examine the characteristics of settlement patterns, architectural remains, sculpted monuments, and ceramic evidence. The early Middle Preclassic (early first millennium BCE) material collected in stratigraphic pits at Tamchén and another locale constitutes the earliest evidence of colonization known so far in a broader central lowland area. From then until the Late Classic period, which was followed by a dramatic demographic decline, the area under study witnessed relatively constant population growth and interacted with different parts of the Maya Lowlands. However, a number of specific and previously unknown cultural traits attest to a rather distinctive regional development, providing novel information about the extent of regional variation within the Maya culture. By analyzing settlement pattern characteristics, inscriptional data, the distribution of architectural volumes and some other features of the currently visible archaeological landscape, which largely reflects the Late Classic situation, we reconstruct several aspects of sociopolitical and territorial organization in that period, highlighting similarities with and differences from what has been evidenced in the neighboring Río Bec region and elsewhere in the Maya area.

## Introduction

**Fig 1:**
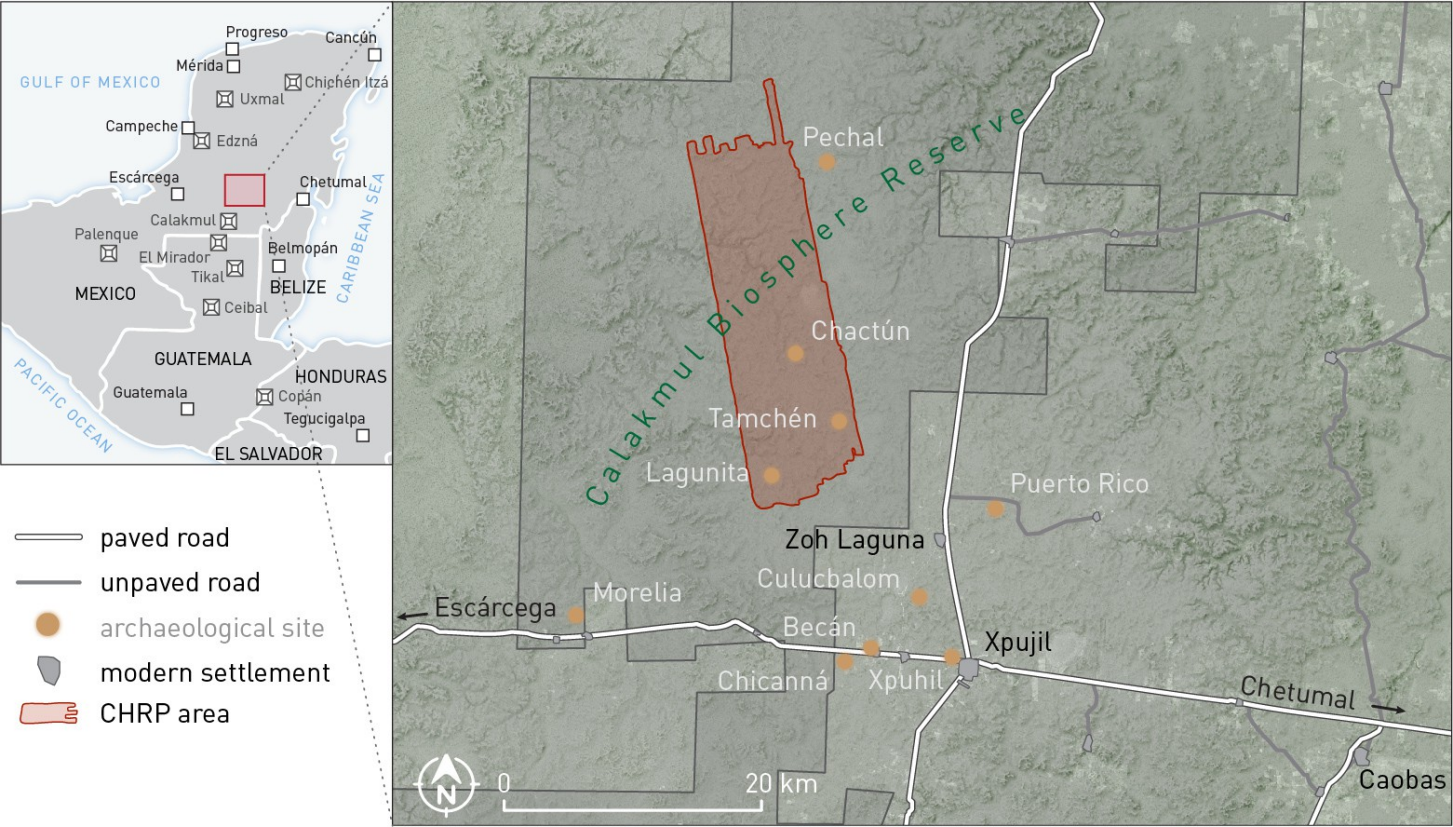
Location of the CHRP area and major archaeological sites in eastern Campeche, Mexico.

Less than three decades ago, some of the largest gaps on the archaeological map of the Maya area were in the central lowlands of the Yucatán peninsula, particularly in the southeastern part of the Mexican federal state of Campeche. From 1996 to 2007, seven field seasons of archaeological reconnaissance were conducted in the southern sector of the now uninhabited Calakmul Biosphere Reserve and the adjacent sparsely populated area extending south of the modern town of Xpujil [1, 2]. In 2013 and 2014, we continued our surveys in the northern part of the Biosphere, which was archaeologically totally unexplored, and located Chactún, Tamchén and Lagunita, three major sites with massive architectural complexes and sculpted monuments [3–5]. Some unexpected characteristics of these sites provoked a number of questions regarding the role of the area in broader cultural processes in the Maya Lowlands. In order to address such questions, in 2016 we acquired full waveform airborne laser scanning (ALS; lidar) data for an area of about 240 km^2^ encompassing the three sites (Fig 1). Field surveys and test excavations were carried out within the Chactún Regional Project (CHRP) in 2017 and 2018.

In a previous study [6], we focused on the techniques of water management and agricultural intensification reflected in specific types of landscape modifications. By analyzing their characteristics, distribution, and chronology, we examined their practical functions, which were of foremost importance in the region characterized by the lack of permanent water sources, and explored the sociopolitical structures involved in their construction and maintenance. We also interpreted the significance of these features in Maya worldview and ritually sanctioned political ideology, discussed their role in landscape construction and conceptualization, and showed their potential for addressing other archaeologically relevant questions, including population estimates, settlement dynamics, and the processes that led to the demise of Classic Maya culture in the central lowlands.

In the present contribution we examine other types of archaeological data, which indicate that the area, although clearly connected with other parts of the Maya Lowlands, witnessed a rather distinctive developmental trajectory materialized in some specific and formerly unknown cultural traits. Based on ceramic evidence, we reconstruct the settlement history and, by analyzing various elements of the currently visible archaeological landscape, which largely mirrors the Late Classic, pre-abandonment situation, we also show that some meaningful inferences can be made regarding the sociopolitical organization in that period.

## Methodology

ALS data were acquired in May, 2016 (at the end of the dry season) by the National Center for Airborne Laser Mapping (NCALM), USA. After their initial data processing (conversion from full-waveform to point cloud data, ground classification) [7, 8], we performed final processing (additional ground classification, visualization). The density of the final point cloud (12.8 average classified ground returns per m^2^ on a combined dataset) and the quality of derived elevation model with a 0.5 m spatial resolution [9, 10] proved very suitable for detecting and interpreting archaeological features with clearly defined minute elevation differences. During ground-truthing we noticed no data collection and processing artifacts (commission and omission errors) in the elevation model, except in two cases where overgrown structural elements on top of a pyramidal mound were classified as vegetation and eliminated, resulting in a deformed shape of the building.

**Fig 2:**
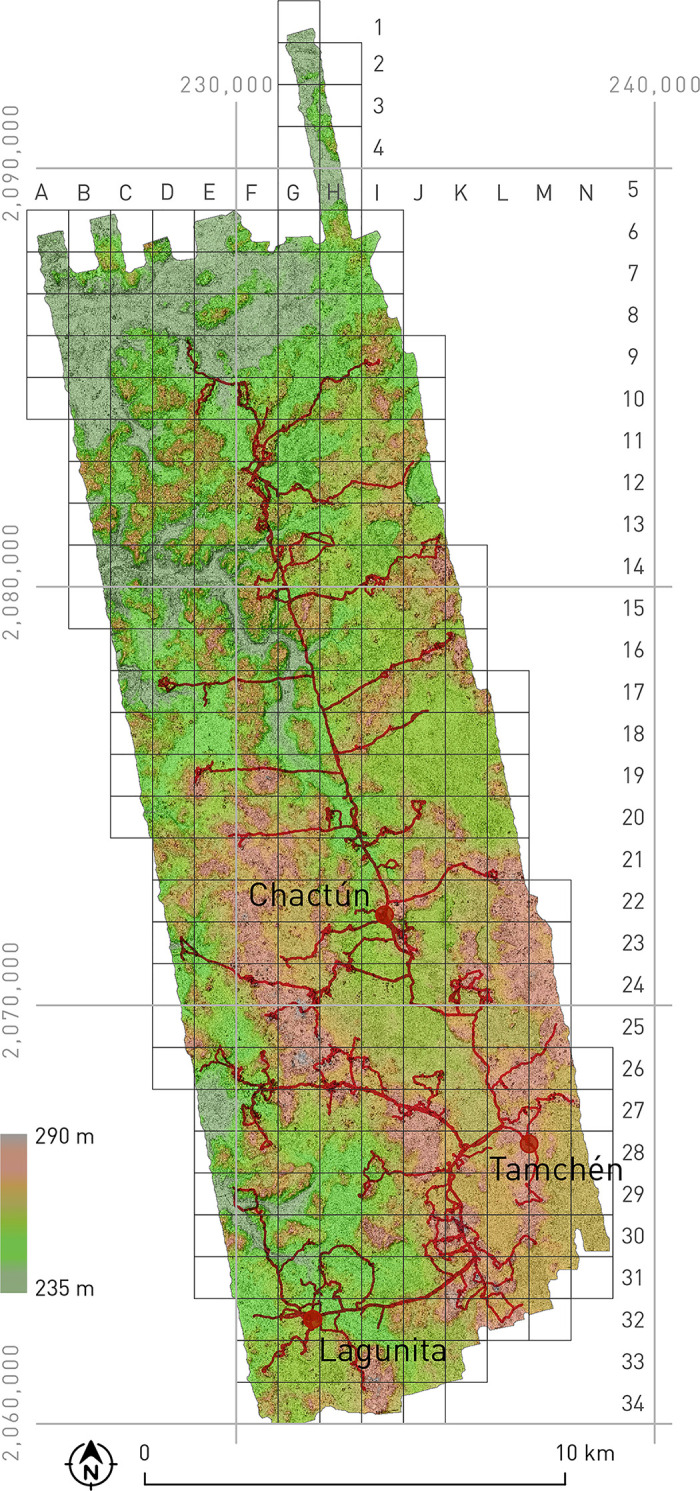
Visualization of the ALS-scanned CHRP area, showing the tracks followed in field surveys and the quadrants used to identify groups of archaeological vestiges.

To annotate polygons of structures and other features, we used Visualization for Archaeological Topography (VAT) created in Relief Visualization Toolbox [9, 11] and computed with settings for general and flat terrain. We found these visualizations well suited for delineating and interpreting archaeological remains. Figs 2, 4 to 6 and 16 to 18 present a combination of flat terrain VAT overlaid by 50% transparent general terrain VAT (combined VAT), while Figs 2 and 4 to 6 also show colored elevations. VAT can be used to enhance the visibility of features of a variety of scales, height, orientation, and form; they can be convex or concave and can sit on terrain that ranges from flat to very steep. Further, the results are comparable across diverse geographical areas, the method does not introduce artificial artifacts, the visual extent and shape of recorded features are not altered, and small topographic features are shown in the same way irrespective of their orientation or shape, allowing us to judge their height and amplitude.

The lack of modern disturbances in the CHRP area, which has been almost uninhabited ever since the demise of the Classic Maya culture in the 10th century, was an important advantage in interpreting ALS data. Even recent activities, related to the exploitation of wood and chicle (sapodilla tree sap for producing chewing gum) during several decades before the foundation of the Calakmul Biosphere in 1989, have left no traces visible on ALS imagery, except for a few long abandoned trails. However, in order to obtain information on time-dependent changes, intensities of occupation in particular periods, architectural details, sculpted monuments or other features undetectable on lidar imagery, pedestrian surveys were accomplished in 2017 and 2018, including collection of surface material and excavation of a number of test pits.

As it was impossible to thoroughly survey the whole area in a reasonable time span, we devised a sampling strategy based on the inspection of ALS visualizations and a preliminary and necessarily rough classification of areas appearing to be representative of the prehispanic cultural landscape: field surveys included all major settlements with monumental architecture (where stone monuments and other vestiges potentially relevant for understanding sociopolitical organization were expected to be found), most of the minor residential clusters, and a number of sectors with different types of landscape modifications (terraces, ridges, water reservoirs, canals, quarries) [12, 13]. Using the Oruxmaps cartographic application, designed for the Android operating system (http://www.oruxmaps.com), and devices including GPS receivers (tablets, phones), we recorded all the tracks followed (Fig 2) and points of interest visited, adding georeferenced photographs and descriptive data, which were then converted into files/layers of the ArcGIS Geographic Information System. The extents of land with different types of archaeological remains and the areas surveyed in the field, considering a 40 m wide zone on each side of the recorded tracks, are specified in Table 1.

**Table 1:** Coverage of field surveys in the CHRP area.

	area	area surveyed	
	km^2^	km^2^	%
Uplands: architectural remains	13.04	4.9	37.6
Uplands: terraces, ridges, quarries, lime kilns	136.83	6.4	4.7
Uplands: unmodified	10.67	2.8	26.2
Uplands: total	160.54	14.1	8.8
Wetlands (bajos): canals, aguadas/reservoirs, ridges	19.35	1.9	9.8
Wetlands: unmodified	58.70	2.6	4.4
Wetlands: total	78.05	4.5	5.8
CHRP area	238.59	18.6	7.8

Based on UTM cartographic projection, the scanned area was divided in 1 km^2^ squares designated with letters and numbers, as shown in Fig 2. Archaeological vestiges of different types were grouped in arbitrary units whose labels correspond to their location in the grid followed by a letter.

For the analysis of architectural volume densities discussed below, volumes were determined using manually recorded boundaries of platforms and buildings of different types. The platforms included in these calculations are evidently artificial flat surfaces that stand out from the surrounding terrain, support other structures, or most likely served this function, even if no upper buildings are currently visible. Agricultural terraces, ridges, causeways, cairns, and the like were not considered. The perimeter polygon around a building or platform was drawn where the interpreter could define the boundary between artificial (modified) and natural terrain. We coded the algorithm for the volume calculation in Python and mainly used the arcpy library. The procedure consists of two main steps. First, the vertices of the perimeter polygon defining the area with structures are interpolated using the natural neighbor technique to obtain a raster surface for each polygon. In the second step, the generated surfaces are used to clip the same areas on the elevation model to obtain raster surfaces of the structures. For each surface pair the cut fill tool of ArcGIS Pro is called to calculate the volume difference between the two; the architectural volume represented by this difference is then attributed to each perimeter polygon. These polygons were converted to points and the volume density (Fig 17) was calculated using the kernel density function in ArcGIS Pro (output cell size: 50 m; search radius: 564 m; area units: km^2^).

### Archaeological characteristics of the area

The CHRP area lies within the karstic Elevated Interior Region of the Maya Lowlands, characterized by a severe lack of perennial surface water and almost no access to the groundwater table [14]. Most of the annual rainfall, which is between 1000 and 1500 mm, is received during the rainy season, from May to December. The uplands are covered with seasonally dry, deciduous tropical forest, whereas seasonally flooded wetlands, or *bajos*, are filled with dense scrub forest adapted to wet and dry extremes. The relief is crisscrossed by a number of low valleys etched by intermittent streams, with the major regional drainage divide running in a north-south direction (Fig 2). Fresh water is only available in a number of small lakes or *aguadas*, typically located along the margins of bajos, but even these water sources largely dry up during the dry season.

**Fig 3:**
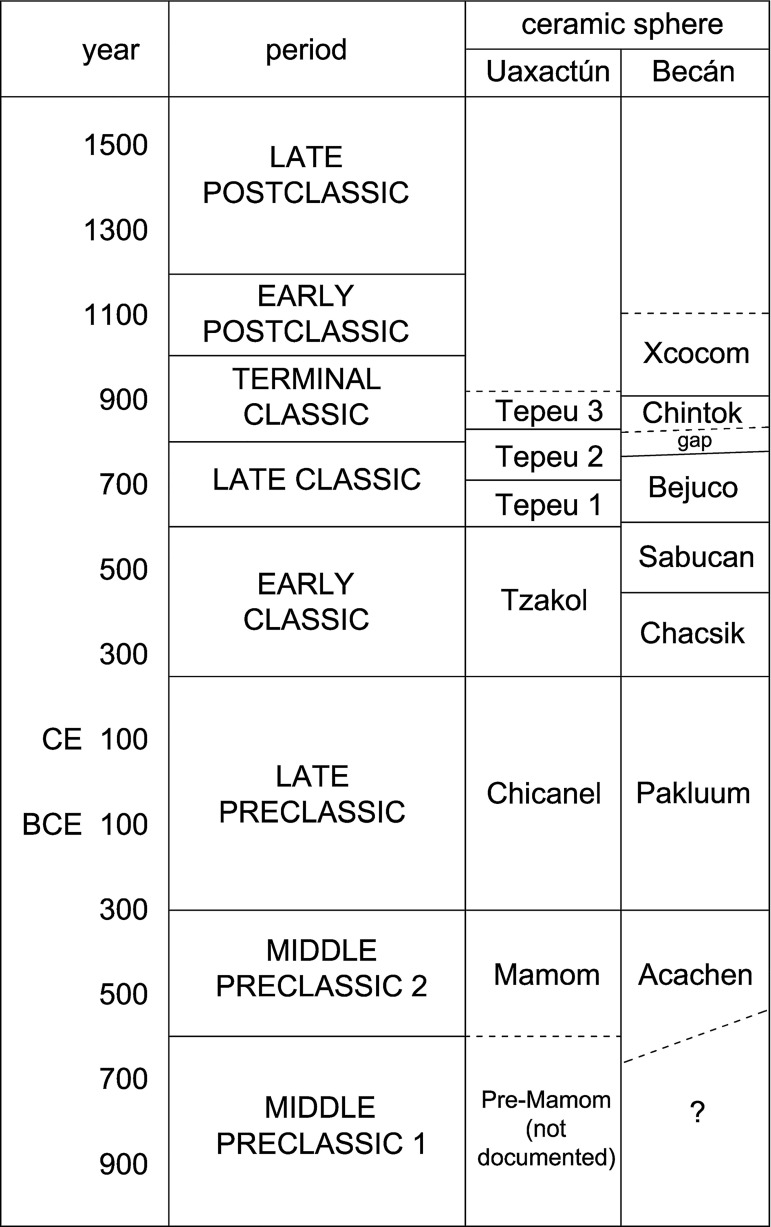
Chronological chart (after [15]).

The archaeological landscape visible on lidar imagery for the most part reflects the Late-to-Terminal Classic situation, a result of accretional cultural processes that largely concluded by the onset of the Postclassic period (see chronological chart in Fig 3). Descriptive data on the archaeological remains documented in the area, the results of test excavations, iconographic and epigraphic analyses of the sculpted monuments, and analyses of soil samples and ceramic and lithic material are presented in the 2017 and 2018 field season reports [12, 13].

**Fig 4:**
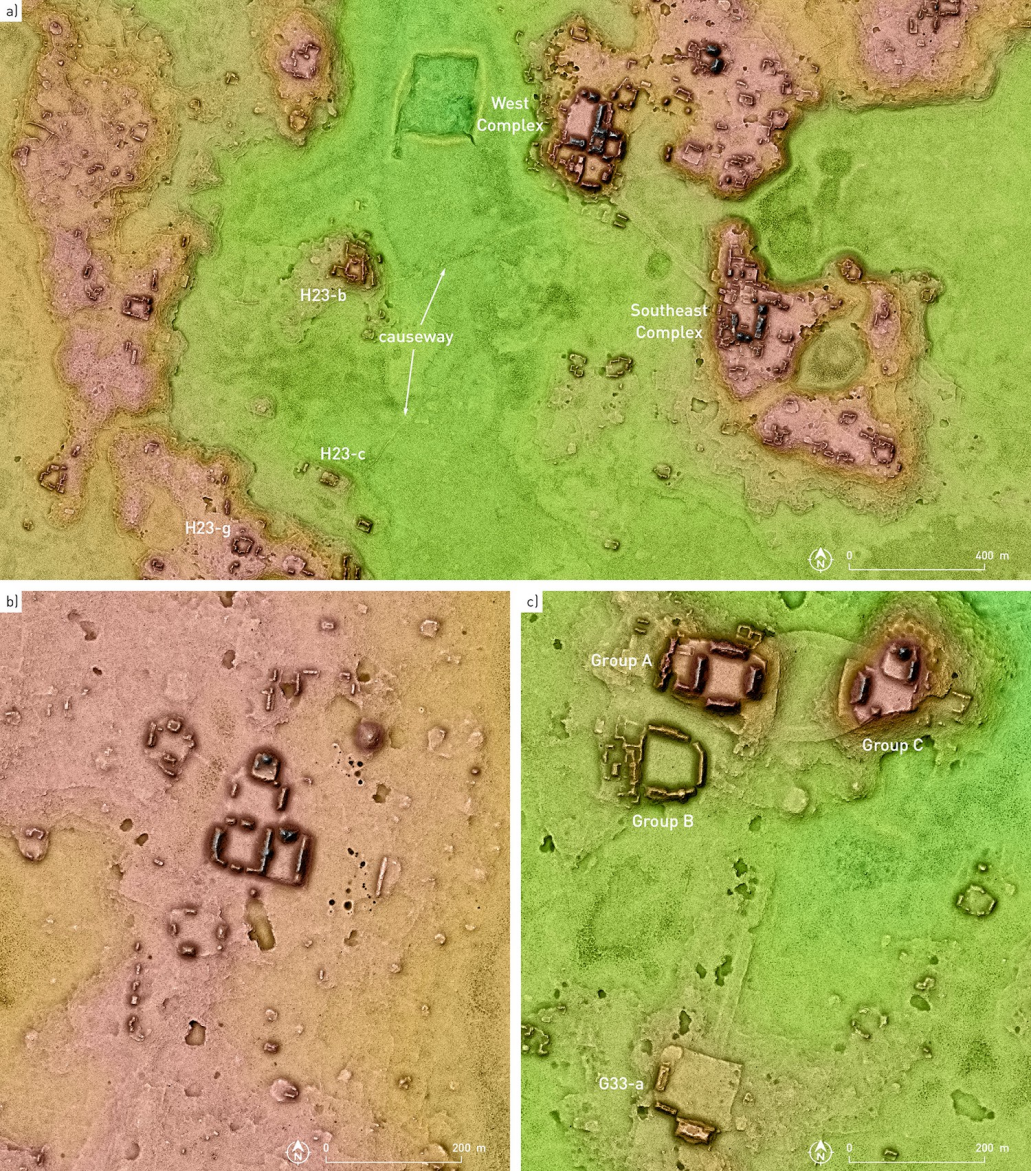
Chactún and the surrounding area to the southwest (A); Tamchén (B); Lagunita (C).

**Fig 5:**
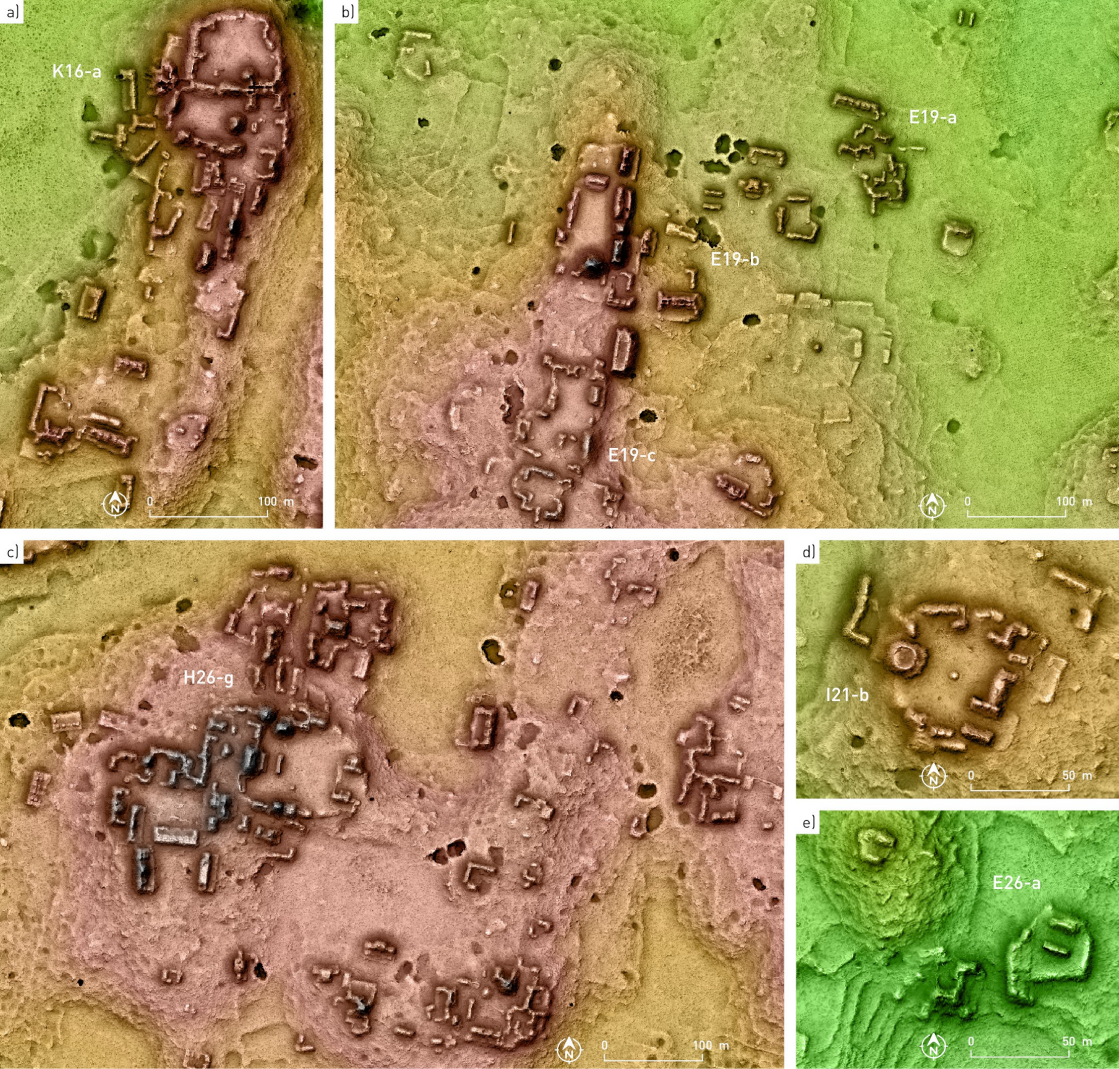
Examples of settlement clusters.

**Fig 6:**
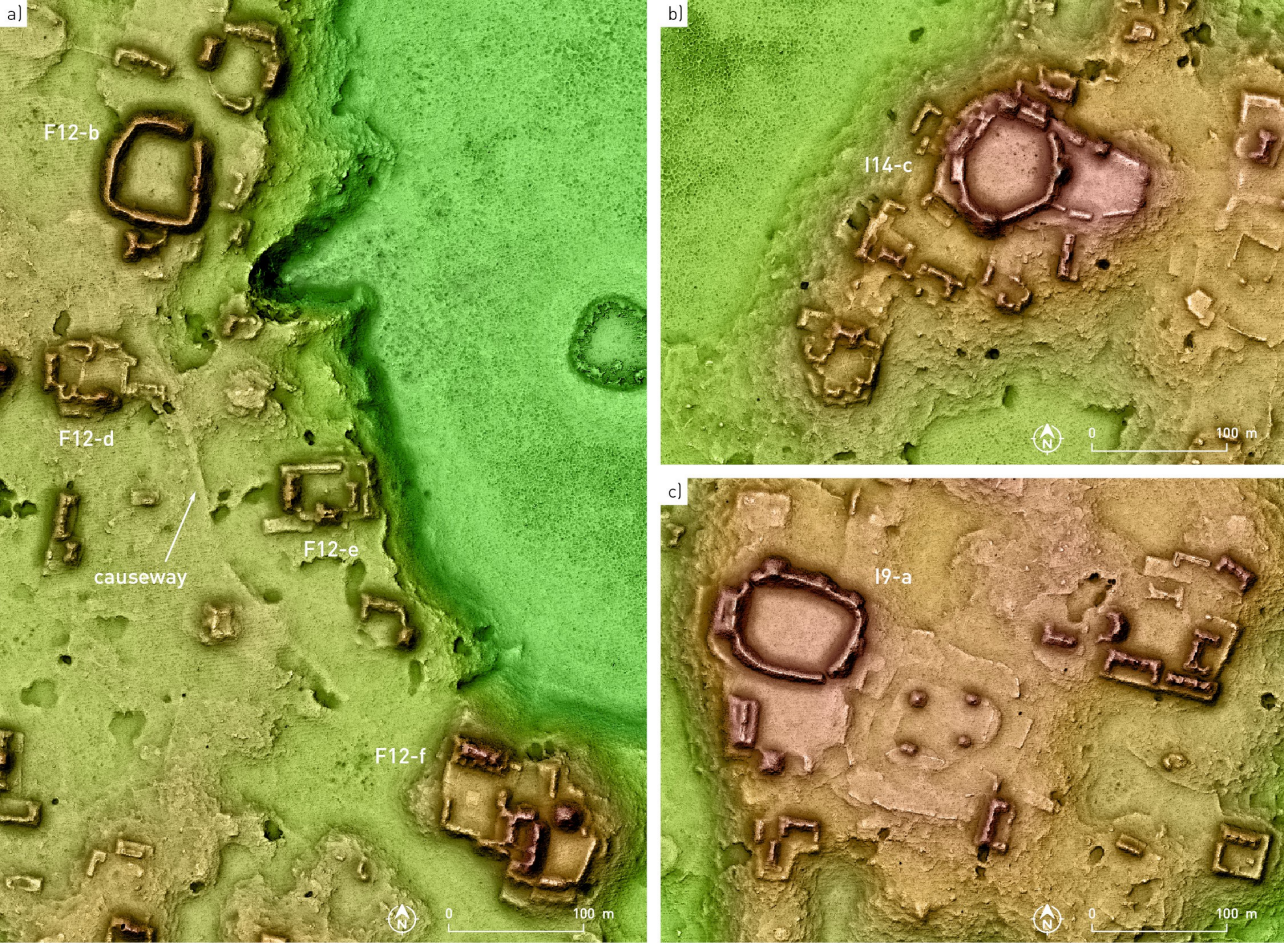
Settlement clusters with Pechal-type plazas.

Aside from complementing the data on the extent and characteristics of the previously mapped Chactún, Tamchén and Lagunita (Fig 4; for detailed information on these sites, see [3–5]), the ALS-derived elevation model revealed a remarkably large number of architectural agglomerations and expansive areas with landscape modifications related to water management and intensive agriculture (Table 1). Since the presence of many elongated or curvilinear, practically continuous mounds (Figs 5 and 6) makes decisions about how many structures they correspond to entirely arbitrary, it is difficult to compare quantitatively the density of architectural remains in the area with other parts of the Maya Lowlands. However, even with a conservative approach, considering many complex mounds as a single structure, we obtained about 61 structures/km^2^, whereas in an extensive area of the Guatemalan Petén, the density based on PACUNAM lidar survey turned out to be 29 structures/km^2^ [16]. As argued elsewhere [6], the surprising density of archaeological remains in the CHRP area reflects a successful adaptation to the less than optimal environmental conditions, although the Late Classic overexploitation of natural resources combined with unfavorable climatic change led to increased vulnerability and disintegration of the complex sociopolitical structures, which finally resulted in a dramatic demographic decline.

The settlement pattern, with a number of architectural groups of different sizes and variable complexity, exhibits similarities with the neighboring Río Bec region to the south [17–19], but also some notable differences. In the area of Becán, the largest Río Bec site (Fig 1), elevated terrain with good drainage was favored for settlement, but steep slopes were avoided [20], and such locational preferences were also observed in the territory extending further south [1]. Similarly, the highly rugged relief, as well as the lack of aguadas, can account for the very few architectural remains in the northwestern part of the CHRP area, but even in that section numerous terraces and ridges can be observed, with occasional low platforms that must have supported perishable structures, probably temporary shelters associated with seasonal field exploitation. The distribution of archaeological vestiges over large extents of the area, including hydraulic and agricultural features, is almost continuous and—in combination with insufficient chronological data—makes it impossible to group them in “sites”, as traditionally defined in archaeology [21: p. 26] and representing discrete spatial units that may have had some significance in prehispanic social organization [22]. The only notable exceptions, which differentiate the area from the overall dispersed settlement pattern with almost no nucleation in the Río Bec region [18], are the three major urban centers of Chactún, Tamchén and Lagunita, which occupy some of the highest prominences. As established by viewsheds computed for all major building tops and based on the lidar-derived digital elevation model, these centers would have been mutually visible.

The buildings, most of which had residential and functionally related uses, are regularly arranged in either patio groups or informal compounds with no commonly shared ground plan, and there are a number of clusters with contiguous patio and plaza compounds (Fig 5). While the architecture composing several groups can be defined as monumental, it is noteworthy that the structures exhibit a continuous range of sizes, without any clear distinction between what may have been simple domestic and palatial buildings. A similar continuity is observed in the sizes of spaces surrounded by buildings, making difficult any meaningful distinction between what could be defined as courtyards or patios, plazuelas and plazas. A special layout is represented by what we refer to as a Pechal-type plaza, a roughly circular space surrounded by almost continuous curved buildings (Fig 6). Such amphitheater-like complexes were first noted at Pechal and Peor Es Nada [23], located about 15 and 20 km northeast of Chactún, respectively.

The shapes of many ruined buildings, in which only the remains of walls that delimited inner rooms are preserved, without any accumulation of material that results from the collapse of vaulted spaces, reveal that they had thatched roofs. However, the remains of vaulted rooms prevail even in minor structures. Aside from a simple rectangular ground plan, which is evident in individual structures, a few special layouts can also be discerned. Quite common is a C-shaped ground plan, sometimes with a supporting platform (Figs 4, 5 and 6). As mentioned above, numerous ruined structures present the shape of elongated, curved or winding mounds, which must have resulted from the collapse of multi-room houses or various buildings placed contiguously, either aligned or at different angles with respect to each other. This is another distinctive feature of the CHRP area, making it impossible to delimit individual structures or households and preventing reliable population estimates based on structure count and common in Maya archaeology (it is for this reason that we employed different methods [6]). Both these residential clusters and elongated standalone structures, which are also common, were often built on supporting platforms (Figs 5 and 6). In many cases only platforms survive, which must have supported structures built of perishable material; their ground plans are rectangular, rounded or entirely irregular. Stairways leading to the buildings’ main entrances are partially exposed in many cases.

**Fig 7:**
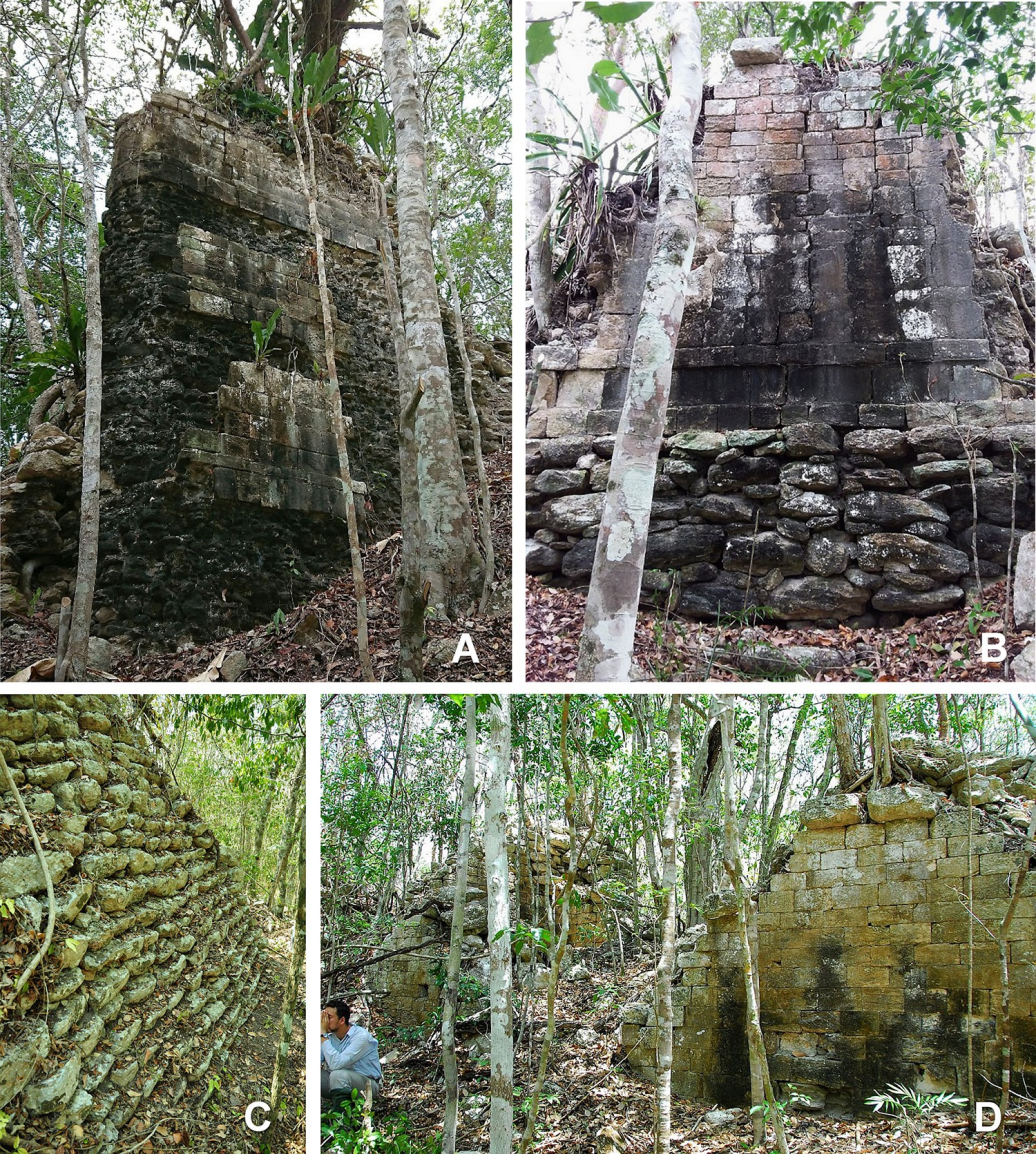
Architectural characteristics: two-tower building in group H31-e, west tower, looking northeast (A); northernmost building of group E19-b, north face with basal molding (B); stepped slope of a pyramidal structure in group K24-b (C); two-tower building in group D23-b, looking northeast (D).

**Fig 8:**
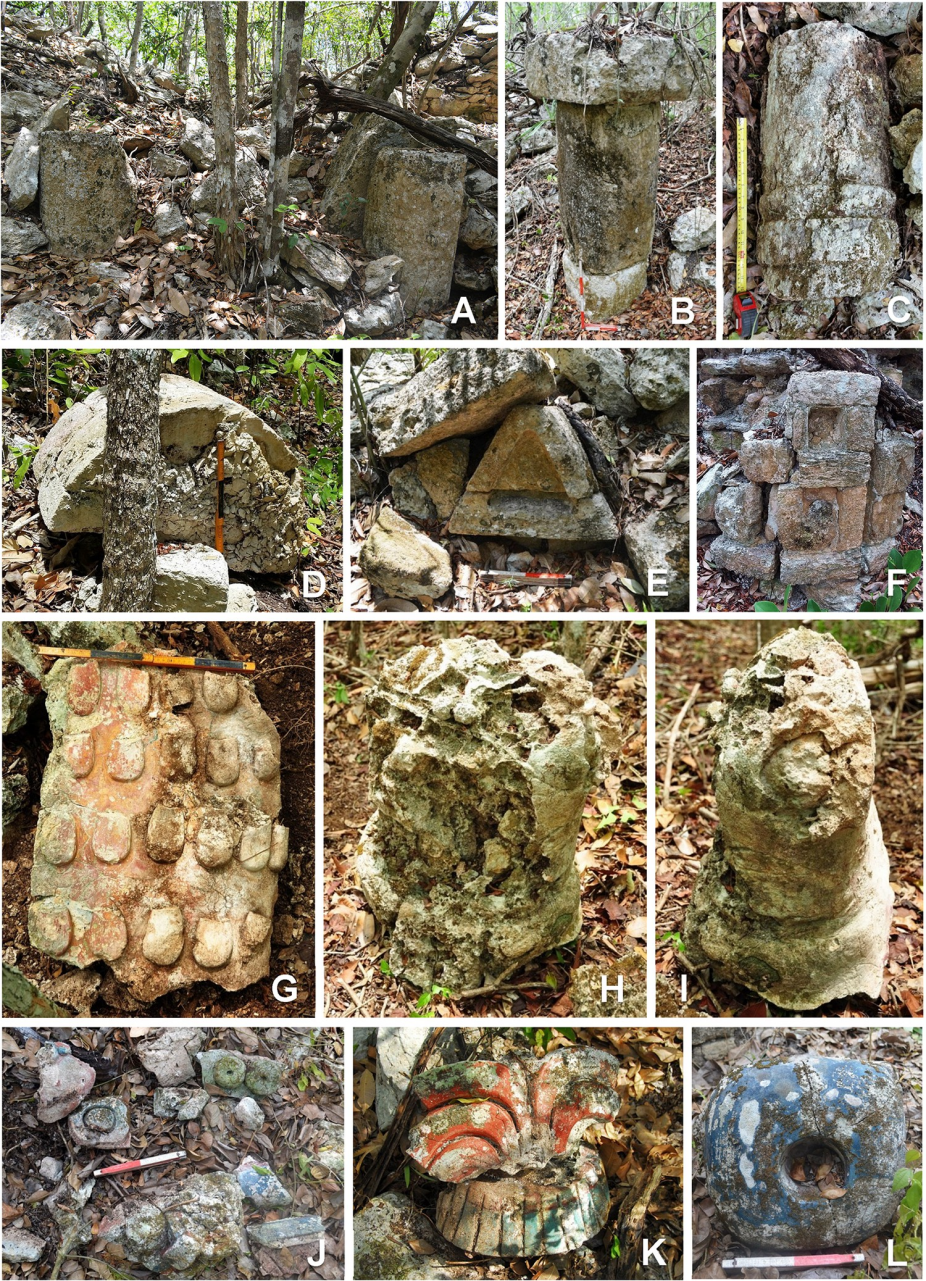
Architectural characteristics: columns in group D23-b (A); column with capital in group E10-d (B); decorated colonnette in group F25-a (C); fragment of masonry column in group F15-a (D); decorative element in group D17-a (E); decorative element in group E19-b (F); fragment of painted stucco decoration in group F15-c (G); human torso covered with painted stucco in group F15-c (H, I; for a similar torso found at Dzibanmac south of Xpujil, see [32]); fragments of painted stucco decoration in group K16-a (J, K, L).

**Fig 9:**
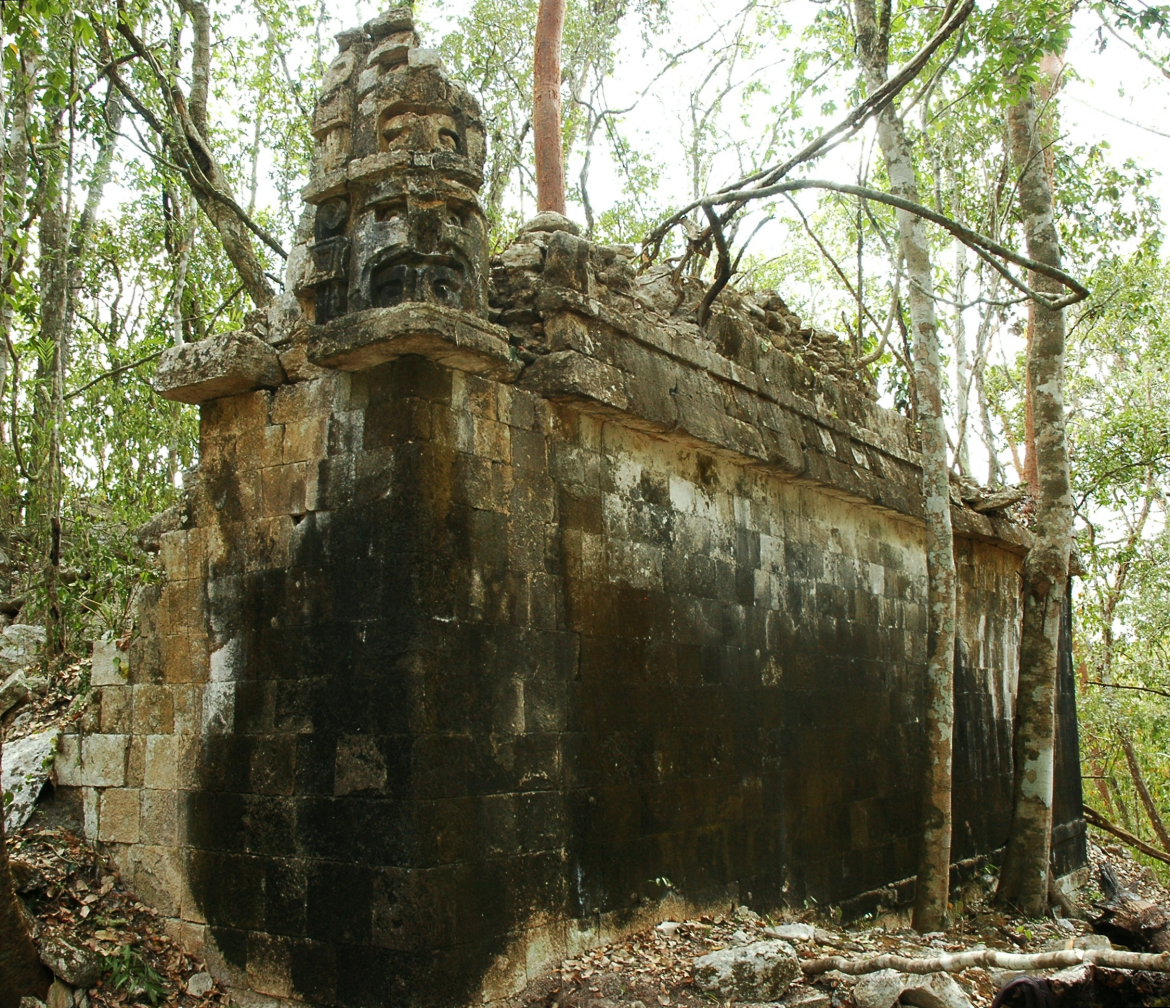
Structure with stacked masks in group F26-b, looking southwest.

**Fig 10:**
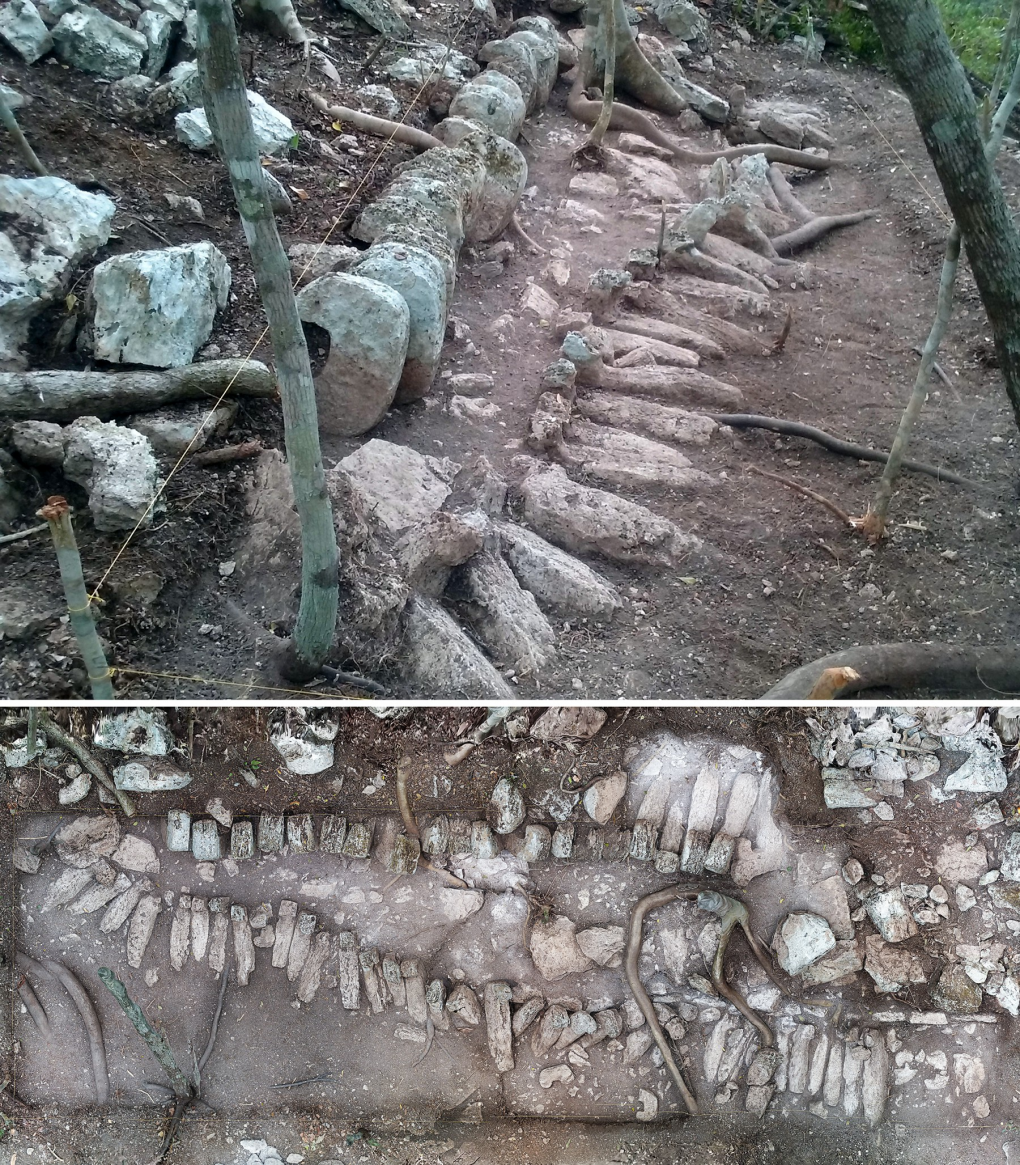
Array of fang-like stones on the north side of the main structure of group G33-a, looking west (top) and from above (bottom).

The CHRP area is located in the territory between the Río Bec and Chenes regions to the south and north, respectively, with their characteristic Late and Terminal Classic architectural styles [24–27]. However, instead of corroborating the previous hypotheses about the existence of a stylistic continuity between the Chenes and Río Bec regions, the CHRP area attests to much more complex and variable relations with the surrounding regions. The Late-to-Terminal interaction with the Río Bec region was intensive and is evidenced in ceramics, in edifices with stepped slopes reminiscent of false stairways, in elongated buildings with tower-like structures at their extremes, and in various elements of architectural decoration (Figs 7–9), the most splendid example being the zoomorphic portal at Lagunita [28]. However, the array of fang-like stones in front of the main structure of group G33-a, an outlier of Lagunita to which a causeway leads from the site core (Fig 4C), is peculiar and its function and significance remain enigmatic (Fig 10) [29]. Both Chactún and Lagunita, which were major Late Classic centers, are characterized by sculpted monuments with inscriptions, true temple pyramids, and massive buildings arranged around large plazas—features that contrast sharply with the neighboring Río Bec sites, where stelae with inscriptions are uncommon, “false” pyramid towers instead of true pyramids are the rule, and architectural groups are typically smaller with fewer structures [18, 30]. Moreover, pyramidal structures, whose sizes range from small shrines to major temples, are ubiquitous in the CHRP area. A common form is a pyramidal structure with two slightly elongated structures attached to its sides and sometimes with a low rectangular platform abutted to its front, but it remains unknown whether these were winged pyramid temples or special types of residence. In contrast to the scarcity of ball courts in the Río Bec zone [31], they are relatively common in the southern part of the CHRP area, even in relatively minor architectural clusters. The ball courts in the Southeast Complex of Chactún and in groups H26-g and H24-e are abutted to a major building (Figs 4 and 5) [12: Fig 3.34].

Some architectural groups are connected by causeways, mostly 3 to 7 m wide, rising less than 50 cm above the surrounding terrain and covered with roughly cut stone slabs (Figs 4 and 6). Much wider are the intra-site causeways at Chactún and Lagunita (Fig 4). The one connecting the West and Southeast Complexes of Chactún is about 30 m wide, large amounts of chert were used in its construction, and a stela-like chert monolith was found along its course. The causeways of Lagunita are about 20 m wide, particularly unusual being the two curved ones that connect Groups A and C [4].

**Fig 11:**
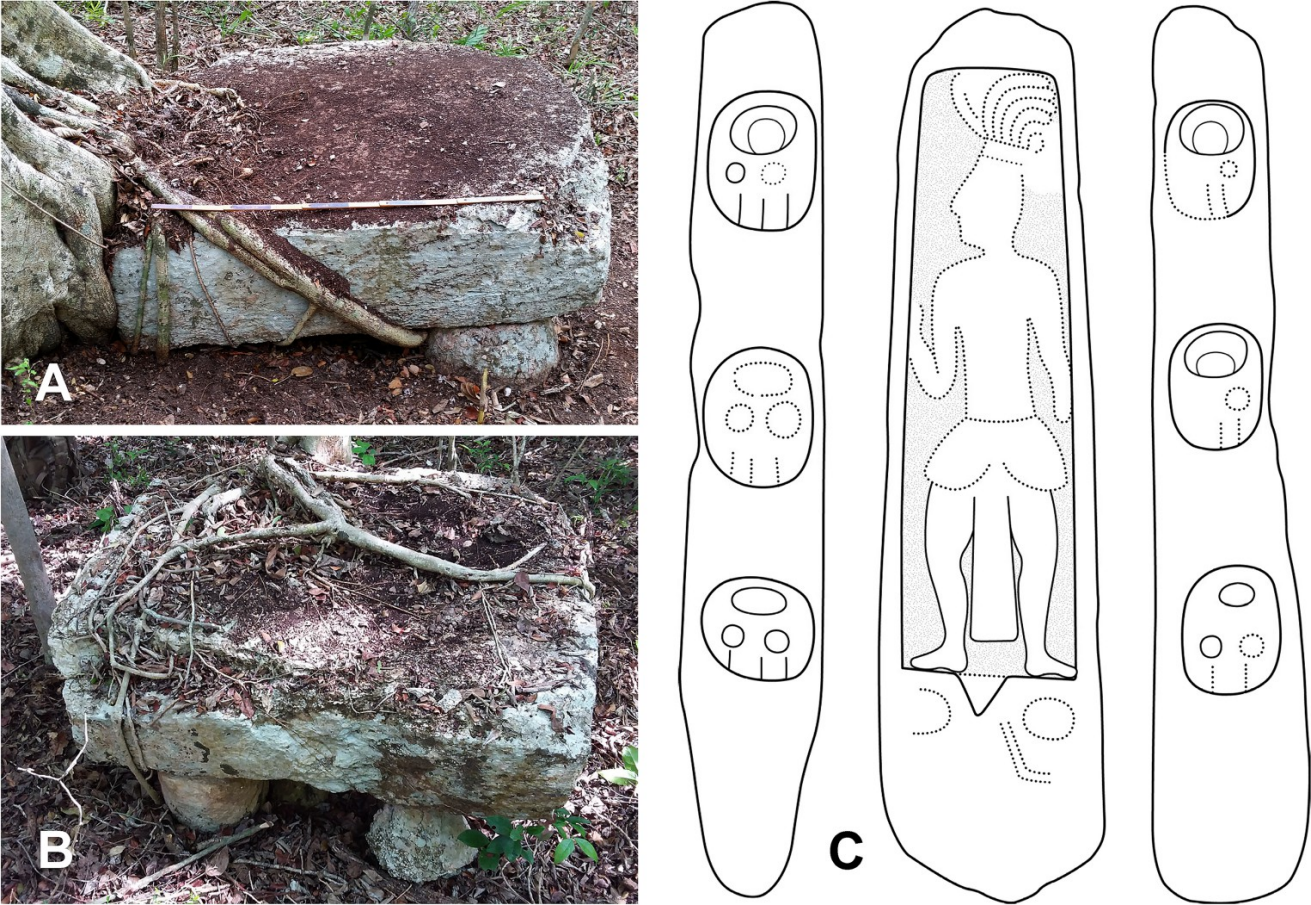
Quadrangular altars on cylindrical supports in groups F10-d (A) and G14-b (B); CHRP Stela 1 (height: 2.08 m, width: 0.52 m, thickness: 0.37 m), showing a local dignitary and a few pseudo-glyphs (C; drawing by Octavio Esparza Olguín).

**Fig 12:**
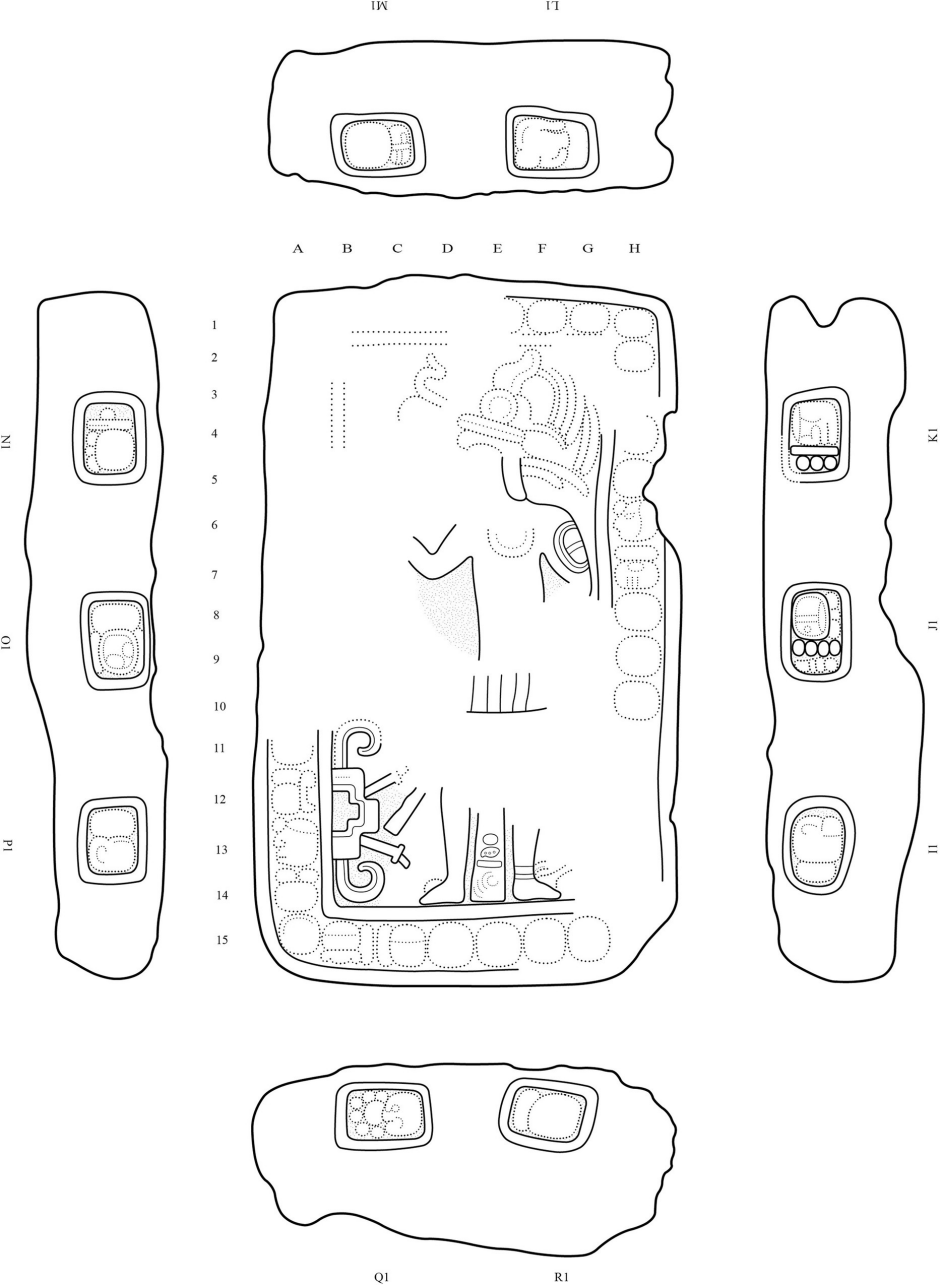
CHRP Altar 1 (length: 1.47 m, width: 0.87 m, thickness: 0.4 m), showing a local dignitary accompanied by a hieroglyphic text, which includes a partially preserved toponymic title (A12-A13) (drawing by Octavio Esparza Olguín).

**Fig 13:**
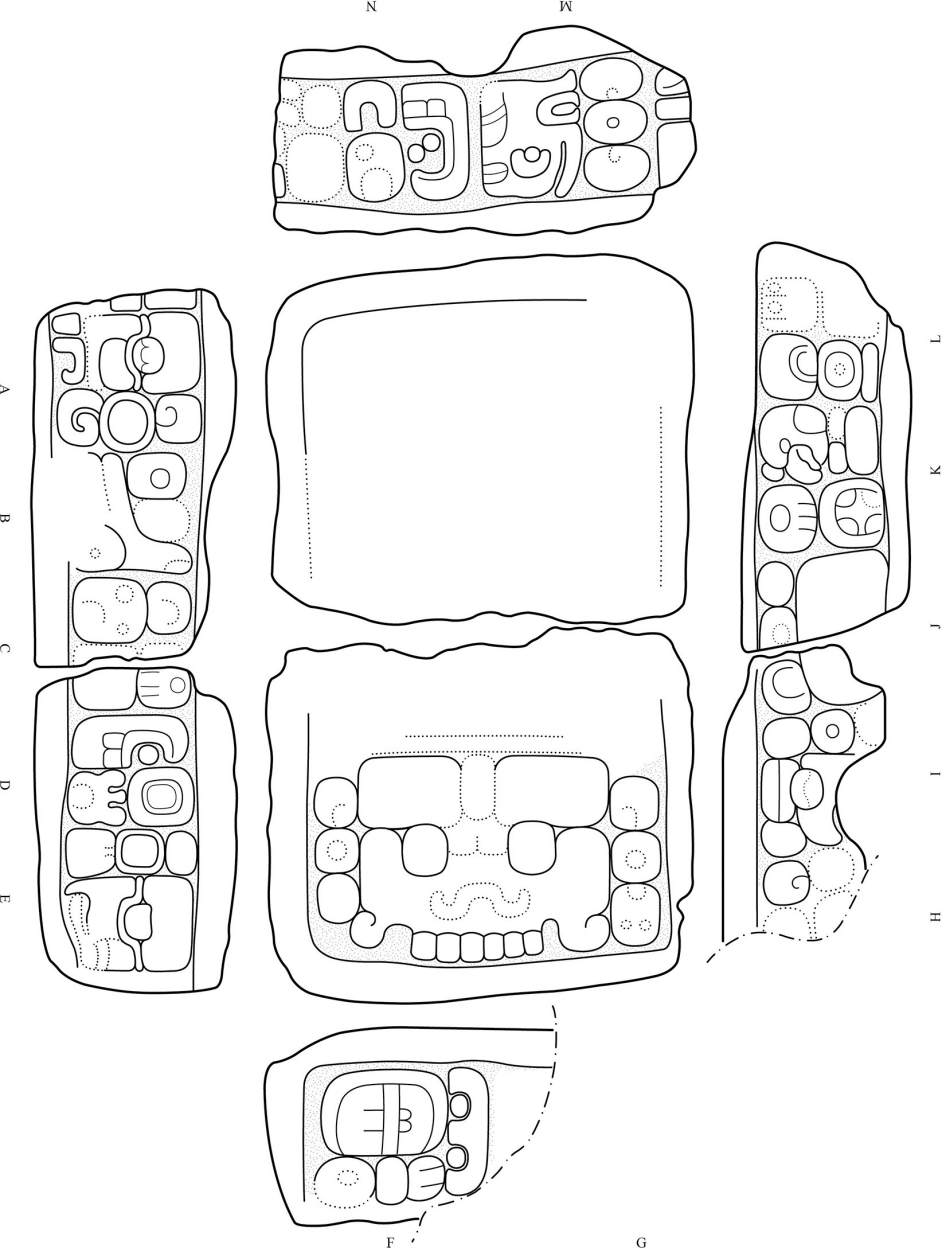
CHRP Altar 2 (length: 1.5 m, width: 0.89 m, thickness: 0.42 m), with a supernatural entity represented on its upper surface and an inscription on its sides, including the *b’aah ajaw* title (K) (drawing by Octavio Esparza Olguín).

**Fig 14:**
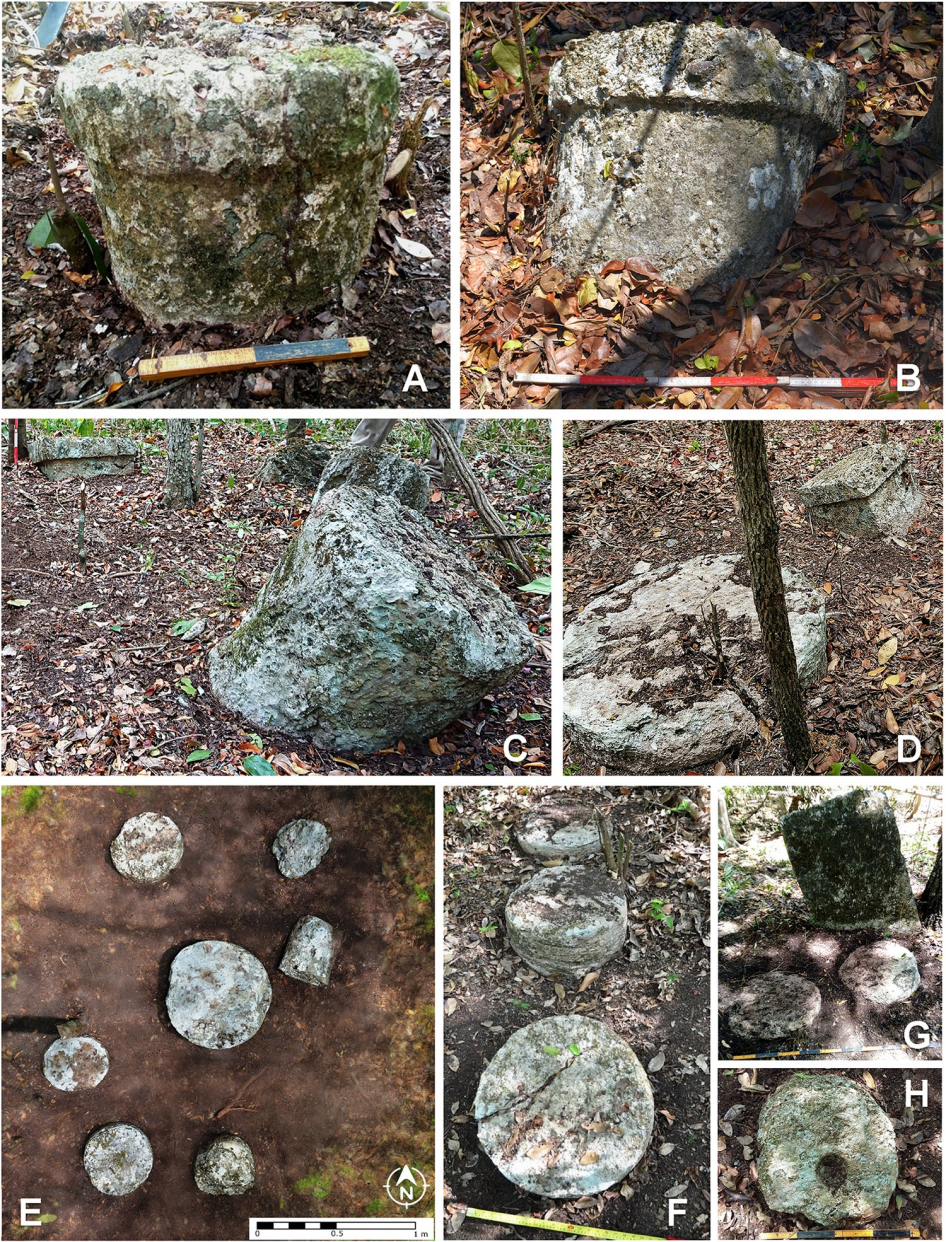
Flanged conical altars and quadrangular blocks in groups H26-c (A), F24-b (B), F26-b (C) and H31-d (D); stone drums in groups F12-f (E) and J14-d (F); plain stela and stone drums in group K30-a (G); stone drum with cavity in group F25-a (H).

Among the many sculpted monuments recorded at Chactún, Lagunita and Tamchén, several stelae and altars have inscriptions and other designs carved in relief. The text on Stela 2 of Lagunita includes an unusual syntax, while Stelae 1 and 14 of Chactún are unique in being covered with glyphs and other designs modeled in stucco [3, 33, 34]; no other such monument has so far been reported in the Maya area. Stelae and altars have also been found in a number of architectural groups throughout the CHRP area. Cylindrical altars have diameters from 1 to 2 m and are up to 0.5 m thick, whereas quadrangular altars are up to 2.2 m long, 1.3 m wide and 0.5 m thick. Several quadrangular altars have cylindrical stone supports (Fig 11A and 11B), representing a regional peculiarity first observed in the case of Altar 1 of Lagunita [33]. While most of these monuments are plain, CHRP Stela 1, found in the plaza of group F15-b, features a local dignitary on the front and a few pseudo-glyphs on its sides (Fig 11C). CHRP Altar 1, a quadrangular monument on four cylindrical supports, with a richly clad local lord on its upper surface and with an inscription on its sides, was found in the plaza of group I14-c, whereas CHRP Altar 2, also quadrangular, was found in the plaza of group H10-b and shows a supernatural being on its upper side and an inscription on its sides (Figs 12 and 13). Another regional peculiarity is the presence of altars in the shape of a cylinder or truncated cone with a flange, or “collar”, which also decorates some quadrangular blocks. They have been found at Lagunita and a few minor architectural groups (Fig 14A–14D).

Finally, small cylindrical stones, less than 30 cm thick and with diameters between 30 and 70 cm, were seen in most of the architectural groups we visited. These stone drums are regularly placed in central parts of plazas and courtyards. Often there is only one, but groups of several, either aligned or forming irregular or roughly circular arrays, are also common; sometimes they are associated with an altar (Fig 14E–14H). Conceivably related to rituals performed by a community or kinship group, they may have served for depositing offerings, as suggested by the cavity in one of them (Fig 14H), but many of them appear to have been reused as construction elements. A number of such stone drums had been previously reported at Pechal and in the surrounding region [23], suggesting that their ubiquity extends beyond the CHRP area.

**Fig 15:**
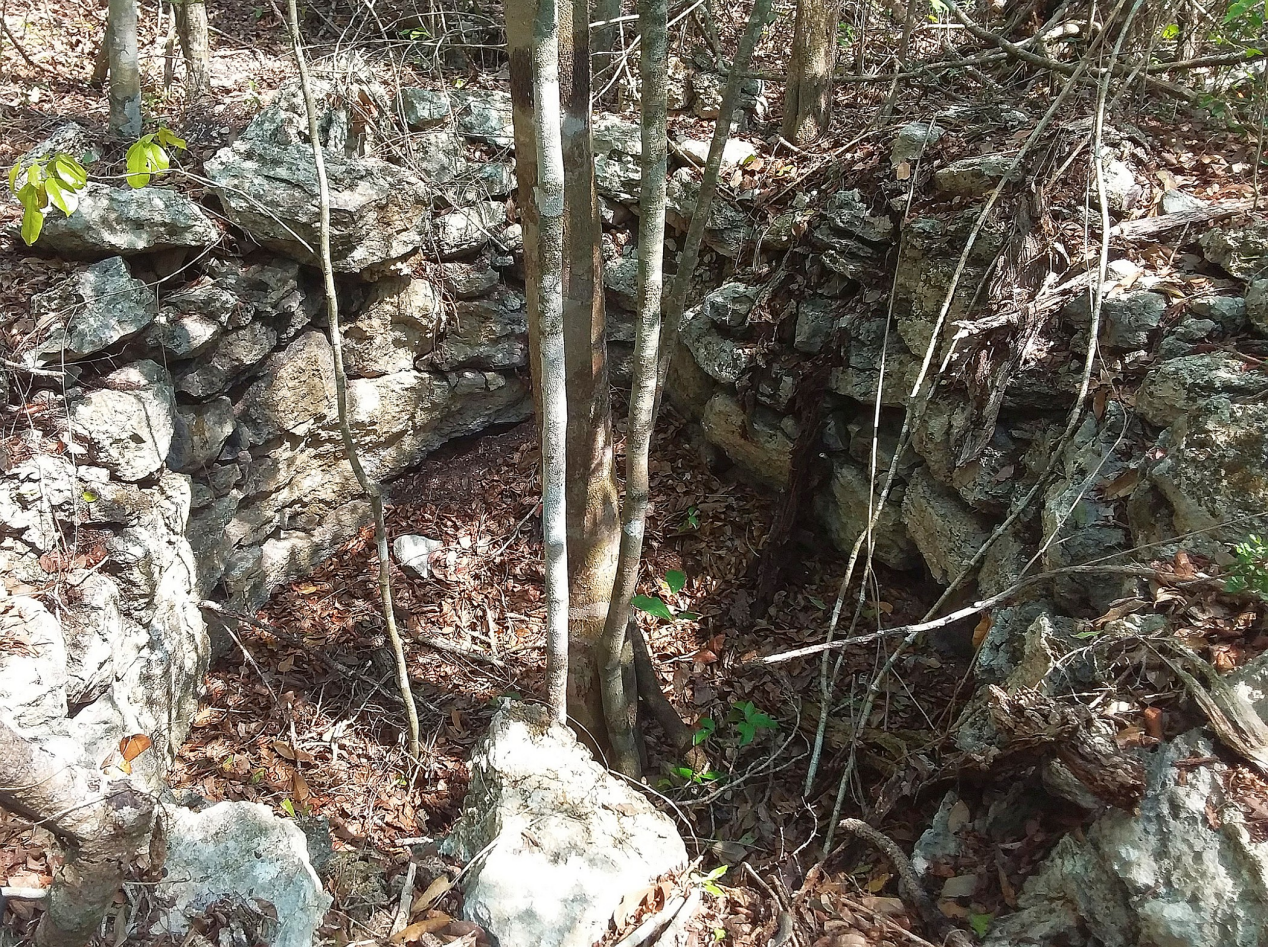
Annular lime kiln in group F11-b.

Apart from the numerous quarries of irregular shapes, many annular cavities delimited by walls of roughly cut stones are placed within or in the immediate vicinity of settlement clusters. They are 2 to 3 m in diameter and currently up to about 3 m deep (Fig 15). Their shapes and the presence of burnt stones observed in several cases clearly indicate that these are remnants of lime kilns, of the type that was common in the northwestern Yucatán peninsula [35–41]. The fact that annular pit-kilns have not been detected elsewhere in the Maya area suggests that different lime production techniques were employed, some of which have left no easily detectable traces [37, 42–44]. Since the enclosed annular kilns required less wood to produce the same amount of burnt lime than open air firing features [38], their abundance in the Puuc and CHRP regions, both characterized by relative scarcity of fuel sources (lower biomass potential due to lower rainfall), is unlikely to be coincidental. While we have inspected 20 annular kilns, many more can be detected on lidar imagery. As in the Puuc region, where the largest number of such kilns has been documented, the CHRP annular kilns are associated with residential compounds and quarries, reflecting the desire to optimize the pre- and post-production transportation efforts [39]. As evidenced by ceramics, the CHRP area had important connections with the northern lowlands, and a test pit excavated in an annular kiln produced Late Classic material [45–47], in agreement with the dating of these features in northwestern Yucatán peninsula [36, 37, 41].

### Settlement history

Due to the postdepositional processes and overgrowth characterizing the tropical environment of the Maya Lowlands, little or no cultural material can be seen on the surface. However, by removing the foliage and about 10 cm of the uppermost layer of soil at a number of spots, mostly in sampling units of 2 × 2 m, we have been able to recover a substantial amount of material, mostly ceramics. Some of it was collected while exposing half-buried stone monuments for epigraphic documentation. Additionally, 31 stratigraphic test pits were excavated. However, given the aims and scope of the CHRP project, focused on surveys based on ALS data, and a limited number of stratigraphic test pits associated with structures, their construction dates can rarely be determined with confidence. Consequently, our ceramic data do not allow us to elaborate a reliable architectural chronology. Neither have we found helpful a comparison with the architectural sequence established for the Río Bec core zone [48], because the similarities in structural types are relatively few or cannot be determined in view of the current state of the CHRP buildings. Nonetheless, and although at many lowland Maya sites, where early occupation layers are buried under later constructions, early material is under-represented in surface collections [49], we believe that the proportions of material from different periods, collected both on surface and in test pits, are reasonably illustrative regarding the population dynamics in the area.

**Fig 16:**
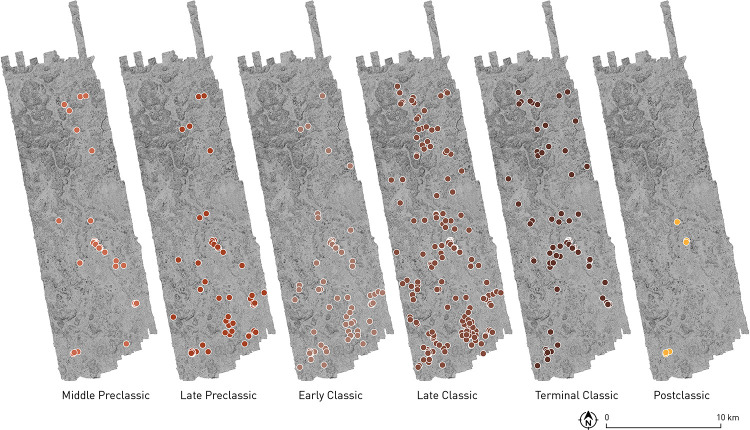
Distributions of ceramics from different periods.

In 2013 and 2014, when Chactún, Tamchén and Lagunita were documented, and during the 2017 and 2018 surveys in the whole area, more than 20,000 ceramic sherds were collected on the surface and in test pits, but only 15,470 were typologically identifiable and chronologically diagnostic. Analyzed by Ball [15, 45] and Dzul [46], they largely pertain to ceramic complexes established in the nearby Río Bec region. Table 2, presenting ceramic frequencies and percentages by period, summarizes the results of these analyses: the numbers and percentages of all identifiable ceramic pieces collected in the CHRP area, both on the surface and in test pits, are compared with those corresponding to surface collections only (3326 identifiable sherds). The differences are attributable to the substantial amounts of early material recovered in Operations L28-1, L28-2 and L31-1 (see below). Fig 16, showing distributions of ceramics from different periods, indicates that, in spite of local variations, there was a rather constant population growth in the area from the beginning of the Middle Preclassic to the Late Classic.

**Table 2:** Ceramic frequencies and percentages by period, corresponding to all identifiable pieces collected both on the surface and in test pits (CHRP total) and to surface collections only (CHRP surface).

	CHRP total			CHRP surface		
	identified sherds			identified sherds		
period	no.	%	cal. %*	no.	%	cal. %*
Middle Preclassic 1	369	2.39	2.58	0	0.00	0.00
Middle Preclassic 2	936	6.05	3.63	92	2.77	1.39
Late Preclassic	2599	16.80	8.24	169	5.08	2.09
Early Classic	3939	25.46	19.63	383	11.52	7.44
Late Classic	6471	41.83	56.45	2001	60.16	68.06
Terminal Classic	1039	6.72	9.06	576	17.32	19.59
Postclassic	117	0.76	0.41	105	3.16	1.43
total	15470	100.00	100.00	3326	100.00	100.00

* Calibrated for different period lengths.

While all Middle Preclassic ceramics in surface collections belongs to the Acachen sphere (Fig 3), the earliest evidence for occupation is the Pre-Mamom ceramics collected in Operations L28-2, in the West Plaza of Tamchén, and L31-1, excavated in architectural group L31-c, a small residential cluster located 3.5 km south of Tamchén (Table 3). In Operation L31-1, the earliest layers contained an offering of limestone spheres [50]; while their meaning is a matter of speculation, similar stone spheres have been found, also in Preclassic contexts, at Ceibal, Nakum, K’axob, and a number of other lowland and highland Maya sites [51–54]. The Pre-Mamom ceramics from Tamchén and group L31-c is related to the Ek ceramic sphere of the northern lowlands. The two localities are the earliest settlements known so far in an extensive area of the central Maya Lowlands [46, 55].

**Table 3:** Ceramic frequencies and percentages by period for Tamchén, corresponding to all identifiable pieces (Tamchén total) and surface collections only (Tamchén surface), and for operations L28-1, L28-2 and L31-1.

	**TAMCHÉN total**			**TAMCHÉN surface**		
	identified sherds			identified sherds		
period	no.	%	cal. %*	no.	%	cal. %*
Middle Preclassic 1	268	12.18	19.60	0	0.00	0.00
Middle Preclassic 2	614	27.91	24.95	25	27.47	16.36
Late Preclassic	1087	49.41	36.14	5	5.49	2.68
Early Classic	46	2.09	2.40	14	15.38	11.78
Late Classic	161	7.32	14.72	30	32.97	44.16
Terminal Classic	24	1.09	2.19	17	18.68	25.03
total	2200	100.00	100.00	91	100.00	100.00
	**TAMCHÉN Op L28-1**		**TAMCHÉN Op L28-2**		**GROUP L31-c Op L31-1**	
	identified sherds		identified sherds		identified sherds	
period	no.	%	no.	%	no.	%
Middle Preclassic 1	0	0.00	268	16.73	101	18.88
Middle Preclassic 2	20	3.94	569	35.52	29	5.42
Late Preclassic	468	92.31	614	38.33	157	29.35
Early Classic	19	3.75	13	0.81	239	44.67
Late Classic	0	0.00	131	8.18	9	1.68
Terminal Classic	0	0.00	7	0.44	0	0.00
total	507	100.00	1602	100.00	535	100.00

* Calibrated for different period lengths.

In relation to the presence of more than 30 chultuns or wells in the very urban core of Tamchén, some of which have unusual depths of up to 14 m and inspired the name we chose for the site (meaning “deep well” in Yucatec Maya: [4]), it is noteworthy that a few of them yielded nothing younger than Middle and Late Preclassic ceramics [15], suggesting that they were used since the initial period of the site. They seem to have resulted from modification of a natural cave system with access to a shallow local aquifer, which may have been seasonally recharged from a nearby bajo to the west (Fig 4B). Since the nearest aguada is relatively far away, about 1.7 km to the northeast, it is very likely that these naturally favorable circumstances had a decisive role in selecting the settlement location, providing an important component of the cosmologically sanctioned political ideology of the ruling elite (for the whole argument, see [6]).

Later Middle Preclassic and Late Preclassic ceramics, represented in samples collected all over the area (Fig 16), has strong affinities to the material in the nearby Río Bec region, northern Petén and northeastern Belize, but also to that of the central western Campeche Gulf Coast and the Northern Lowlands. Tamchén may have been the only major Preclassic center in the CHRP area. Its florescence in that period is attested by both ceramics and monumental architecture, which includes a triadic group. While both Middle and Late Preclassic material was also recovered at Chactún and Lagunita [45], the architecture visible at both sites does not exhibit any typical Preclassic traits [4].

The Early Classic witnessed a population increase throughout the area and a significant occupation at Lagunita, while Tamchén suffered a decline. During the Late Classic period, Lagunita continued to thrive and Tamchén experienced some recovery, but evidently played no major role in regional hierarchy, in which Chactún seems to have achieved a prominent role, with influences from the Petén manifested in both ceramics and architecture [4]. The Long Count dates recorded on the monuments of Chactún and Lagunita, falling in the first half of the 8th century [33], correspond with the period during which the power of Calakmul’s Kaan dynasty declined significantly due to defeats suffered at the hands of Tikal, in 695 and 736 CE. If these events triggered a process of political decentralization and fragmentation in the central lowlands that made possible the strengthening of small local dynasties—as also appears to have been the case at Oxpemul [1]—these developments may well have also contributed to an increased political power of Lagunita and Chactún. Ball [15] suggests that, as a consequence of the demise of the Kaan, contesting forces from the Peten may have moved northward, either precipitating the abandonment of Becán around 730 CE, or taking advantage of the vacuum created by the collapse of this Río Bec regional center.

It was probably around 750 CE when the whole CHRP area reached its maximum population density; according to our estimates based on agricultural potential and needs, and on wood and water availability and requirements, about 15,000 people may have lived in the area at that time [6]. The ceramic material is affiliated with regional spheres common in the Río Bec zone, but connections with northern types persist [15, 45, 46]. Relations with the Río Bec region are also evident in the architecture, in spite of regional peculiarities summarized earlier. A typical example is Structure A-7 of Lagunita, which features a zoomorphic or monster-mouth façade and may have also had two towers at its extremes [28]. A few other two-tower structures have been recorded (Fig 7A, 7D). If the one in group H31-e was built at the end of the Late Classic, as suggested by the ceramics collected in three test pits [46, 50, 56], it was contemporary with such structures in the Río Bec zone, largely dated to the late 8th and the 9th centuries [48]. Also relatively common in the CHRP area are remnants of architectural decoration composed of stone mosaics, colonnettes and other elements characteristic of both Río Bec and Chenes architectural styles (Fig 8).

Population decline after 750 CE can be attributed to a combination of environmental problems and demographic pressure: we have argued that, due to soil depletion, many areas previously adapted for farming could no longer be used and more forested portions of land were cleared for the same purposes, resulting in critically reduced wood supplies; the situation was additionally aggravated by prolonged droughts in the 9th and early 10th centuries [6]. This scenario is supported by social disruptions and ideological changes evidenced in the monuments of Chactún and Lagunita. At some time after their original erection, several stelae and altars were modified or broken and reused in secondary situations [4, 57]). The timing and circumstances of their resetting remain open questions, but it is conceivable that it occurred during the Terminal Classic in conjunction with the arrival of émigrés from the northwest or north of the peninsula. At Becán, a new, immigrant populace unrelated to that associated with the original local ceramic and architectural traditions resettled and renovated the center in the first half of the ninth century [58]. Ceramic and other artifactual evidence has long suggested that they came from the northwestern or northern part of the peninsula [59–62] and were responsible for a revival of the local Río Bec architectural style and an archaeologically visible respect for and veneration of relict stelae and other monuments manifest in the deposition of incense burners and other artifacts at their locations [58, 59]. Chactún may have witnessed similar processes. If the 751 CE date—recorded on Stelae 1 and 12 and the latest known so far from the site—corresponds to the final years of its Petén-related florescence and presaged a possible subsequent abandonment of the site, the desecratory smashing of many monuments could well be related to the arrival of foreign groups of northern origin, whose presence at the site is attested by the occurrence of Terminal Classic Xcocom ceramics.

As indicated by ceramic frequencies, the population decrease in the CHRP area, which started in mid-8th century and continued during the Terminal Classic period, was followed by a dramatic demographic decline (for similar developments in the Río Bec zone, see [48]). The only available evidence of the drastically reduced and impoverished human groups that remained in the area during the Postclassic are fragments of Chen-Mul censers and other ceramic vessels, vestiges of ritual activity at a few monuments of Lagunita and Chactún and in a peripheral group of the latter, and an arrow head associated with a stela at Tamchén [4, 12, 13].

### Late classic sociopolitical organization

Given the CHRP population peak during the Late Classic, most of the currently visible structures were very likely built in that period, even if many of them may have covered earlier constructions. With the limited amount of chronological data and in the absence of reliable architectural chronology, we could only speculate about the sociopolitical organization in earlier periods. However, some inferences in this respect can be made for the Late Classic period.

**Fig 17:**
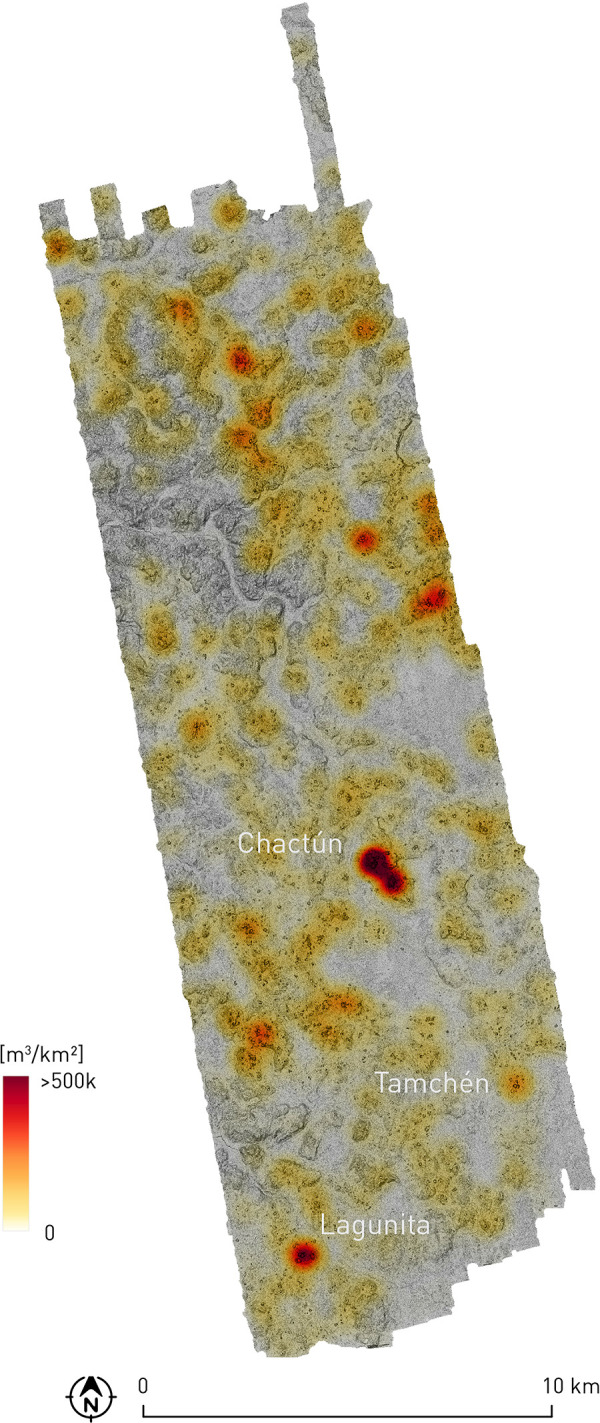
Architectural volume density distribution.

Among several methods that can been used to assess the extent of economic and social inequality, by measuring the distribution of wealth among segments of a particular population, the Gini index has often been applied in archaeology, by using material proxies such as grave goods, artifact distributions and, most typically, household sizes expressed as areas or volumes [63–66]. However, given the scope of our research and the nature of the data we have been able to collect, we consider that none of these approaches would lead to meaningful results, not even the method based on the sizes of residential units. On the one hand, a characteristic feature of the CHRP settlement pattern is the presence of long, often irregularly curved mounds, which evidently resulted from the collapse of contiguous structures, making it impossible to discriminate individual houses. On the other—and in contrast to the situation at Caracol, for example, where households could be defined as discrete spatial (plazuela) units [63]—the CHRP patio or plazuela groups are of highly variable sizes and with differing numbers of structures; they are often contiguous, composing larger settlement clusters, and there is hardly any clue as to which or how many structures made up a single, socially significant group (such as a nuclear or extended family). Neither have we attempted to classify structures according to their hierarchical ranks (for examples of such a procedure, see [18, 49]): since the sizes of buildings and compounds represent a continuum, with no clear-cut distinction between the spaces occupied and used by commoners and those pertaining to the elite (the only exceptions are Chactún, Tamchén and Lagunita), any such ranking would be inevitably arbitrary and the results of analyses would not be particularly meaningful. However, some characteristics of the Late Classic social and political structures are reflected in the distribution of architectural volume density, which is directly proportional to the number and size of the buildings (Fig 17; for the method of calculating volume densities, see above: Methodology), as well as in other evidence discussed below.

While the most pronounced volume concentrations characterize Chactún and Lagunita, which were the only two major Late Classic centers, other areas of greater volume density can be interpreted as material remnants of communities or neighborhoods of different sizes. Variable definitions of both terms [67–69] are irrelevant for the present purposes, since neither the extents and boundaries of these building clusters nor their social correlates can be reconstructed with the available data. Given the characteristics of building remains, their spatial distribution, and the lack of other pertinent evidence, none of the criteria proposed by Smith [70] for the archaeological identification of neighborhoods or districts (larger units composed of several neighborhoods and often with some administrative functions [71]); can be convincingly applied for analytical purposes. Arnauld et al. [72] analyzed the Río Bec social structures through the model of House societies, previously employed in the studies of Maya social structures by Gillespie [73] and similar to the concept of conical clans [74]. A house or a conical clan is considered to be an internally ranked corporate residential unit based on factual or postulated kinship ties and, particularly, on economic cooperation related to agricultural exploitation, craft specialization, or other objectives. In the Río Bec zone, spatial associations of monumental and simpler structures, the differing internal characteristics of particular buildings, and residential clusters of different complexity reflect status distinctions both within and between social groups [72]. To judge by the characteristics of the built environment, such an organization, comparable to the historically documented Late Postclassic situation in the northern Yucatán peninsula [75, 76], is likely to have been prevalent also in the Late to Terminal Classic CHRP area, although some differences must be pointed out. In the Río Bec region, Lemonnier and Vannière [77] were able to determine agricultural production units, each with a household unit and surrounded by terraces, ridges and rockpiles, but they also observed a common association of these features with elite residences. Such spatial relations are also frequent in our case, but many terraces and ridges are distributed continuously over large expanses of terrain, often quite far from residential groups. Low platforms, which must have supported perishable structures, are often scattered within these areas, but there are no clues as to the household or neighborhood to which they may have pertained [6]. A similar situation characterizes northwestern Belize, where agricultural features are spatially discrete from residential areas: Kunen [78] suggests that they constitute concentrated zones of resource control, associated with multiple houses that formed farming communities.

Among other characteristics of the CHRP area, a remarkably large number of vaulted structures should be mentioned. Vaulted rooms prevail even in very modest buildings, which means that the technology involved, requiring much greater resources in terms of manpower, materials and abilities than thatching [39], was shared by or accessible to relatively wide sectors of population, as has also been observed in the Late Classic Puuc region [79]. In contrast to what has been noted elsewhere, e.g. at Caracol [80], a vaulted structure in the CHRP area does not indicate a higher status of its occupant. Furthermore, many buildings, even in minor groups, exhibit fine masonry, entrances with columns, fragments of stone mosaics and other decorative elements of facades, elaborated in both stone and stucco (Figs 7 to 9); these features also suggest a relatively high level of social welfare, similar to that prevailing in the Río Bec region and perhaps comparable to what has been termed “symbolic egalitarianism” in the case of the Late Classic Caracol [81]. Whereas in the rest of the Maya Lowlands ball courts generally characterize major sites, their occurrence in several insignificant architectural groups in the southern part of the CHRP area (Fig 5) implies participation of broader social strata and a weaker political centralization [82–84]. Similarly, the Middle Preclassic ball courts in northwestern Yucatán occur at sites that lack signs of significant social stratification, suggesting that ball game was not always and everywhere an elite political or ritual activity [85]. Finally, the numerous lime kilns in the CHRP area are regularly associated with minor architectural groups; such a distribution of these features in the Puuc region was interpreted as reflecting decentralized lime production organized by small-scale corporate groups [39].

While some of the elements discussed above represent distinctive features of the CHRP area, it is particularly the presence of two Late-to-Terminal Classic urban centers that suggests a rather different sociopolitical organization from that in the neighboring Río Bec region, where the only major site that maintained a prominent role during that period was Becán, but its political sway apparently did not reach the core zone of Río Bec sites. While some Río Bec architectural groups, particularly Kajtún, can be defined as sociopolitical foci, they were largely abandoned before the Late Classic onset of the typical Río Bec phenomenon, characterized by a lack of nucleation [17–19]. In contrast, both Chactún and Lagunita experienced no such abandonment, but rather reached their apogee in that period. As argued in a previous study [6], the lack of agricultural features in the immediate neighborhood of Chactún and Lagunita bespeaks of their power to extract the necessary provisions from surrounding areas and smaller communities; centrally coordinated efforts and standardized planning controlled by the ruling elite are also reflected in the size and shape of the largest rectangular water reservoir at Chactún (Fig 4A). However, various lines of evidence discussed below indicate decentralization trends that led to a relatively complex sociopolitical organization in the Late Classic period.

Whereas Chactún and Lagunita boast a number of elaborately carved stone monuments [33], stelae and altars also are found in many small and insignificant architectural groups. Most of them are plain and of modest dimensions, and possibly analogous to the conical stone altars found in many residential groups in the Puuc region, which may designate the bases of lineage groups [79, 86]. However, two monuments with reliefs were found in groups with no structure higher than two meters; CHRP Stela 1 shows a dignitary on the front and some pseudo-glyphs on its sides (Fig 11C), while the inscription on CHRP Altar 2 includes the title *baah ajaw* (“first” or “main lord”), corresponding to individuals that seem to have been subject to, but not members of, the royal lineage (Fig 13) [87: p. 70]. On CHRP Altar 1, however, which is more elaborately carved, a richly clad human figure is represented on its upper surface, whereas the inscription on its sides includes a toponymic title, which is only partially preserved, but the **AJAW** component clearly indicates that its holder had, in comparison with the protagonist of the other altar, a higher position in regional hierarchy (Fig 12) [88]. Accordingly, this altar was found in the main plaza of group I14-c, a major architectural compound located 8 km north of Chactún (Fig 6B).

Epigraphic evidence from different parts of the Maya Lowlands suggests that, during the Late Classic period, new political strategies were adopted, which incorporated greater numbers of non-royal nobles and high-ranking commoners into the affairs of state and included the creation of new offices and titles, granting subordinates the right to commission their own monuments [76, 87, 89, 90]. Likewise, the evidence presented above indicates a complex sociopolitical organization in the CHRP area, with several levels of decision-making and the presence of lower or intermediate elites with differential access to wealth and power. However, the presence of major seats of power and the hierarchical relations attested in the inscribed monuments referred to above reflect important differences in comparison with the nearby Río Bec region. The fact that the elite architecture beyond these centers is less elegant and monumental than the one characterizing many constructions in the Río Bec region may additionally signal their regional political power, reflecting their ability to impose constraints on their clients. The evidence presented thus suggests that the Late and Terminal Classic sociopolitical organization in the CHRP area was less acephalous than in the coeval Río Bec core zone, which apparently remained beyond the immediate control of Becán.

By the Late Classic, Tamchén had long lost its political importance. In that period, the only major CHRP centers were Chactún and Lagunita, but one part of the area may well have been dominated by Pechal, located to the northeast (Fig 1). In his comprehensive study of the Classic Lowland Maya politics, Martin [87] shows that the inter-polity hierarchy was defined by personal relations between patron and client ruling elites. As he argues, “the evidence consistently supports a hegemonic system, one in which an enduring multitude of polities were arranged within waxing and waning hierarchical orders. Although they were in active competition with one another, there was little or no territorial consolidation or the permanent eradication of rivals–indeed the number of identifiable polities only increased as time wore on” [87: p. 383f]. In the absence of relevant inscriptional evidence, we have no information about the nature of political relations between Pechal, Chactún and Lagunita, let alone about the time-dependent changes in regional political geography. Nonetheless, if the currently visible archaeological landscape represents a materialization of the very late, pre-collapse situation, as the available data indicate, it allows us to make some inferences at least for that specific period, regarding the approximate extent of land that each of these centers controlled.

In the territory extending between Chactún and Lagunita, a zone of diminished architectural volume density can be observed in Fig 17, perhaps indicating a boundary between the two polities. The causeways leading from Chactún to the southwest also suggest its close connections to the settlement clusters in that direction. From the ball court at southeastern extreme of the West Complex, a causeway running across a bajo reaches a peripheral group H23-b. Another one runs from group H24-g to the northeast, passing through group H23-c; we were unable to follow it in the field along its total length, but it likely also reached the West Complex of Chactún (Fig 4A). However, we have no other clues as to the nature of political relations between Chactún and Lagunita. In the absence of epigraphic data and fine-grained chronology for each site, we do not know whether they were autonomous rivals or allies, or perhaps in an asymmetric relationship, not even if they reached their apogee during the same time span. The structure abutted to the southern portion of the zoomorphic portal of Lagunita, evidently added sometime after the original construction of the façade, suggests the existence of lateral towers, which are typical of Río Bec architecture and were often attached *a posteriori* [28]. Since Taladoire et al. [48] argue that structures with towers at Río Bec and some neighboring sites do not appear until the second half of the eighth century, becoming particularly common in the ninth century, the construction at Lagunita might indicate that the site continued thriving after the decline of Chactún, which may have started in mid-8^th^ century, to judge by the latest date inscribed on its monuments, falling in 751 CE. But on the other hand, the only known date from Lagunita is earlier, corresponding to 711 CE [33], and the percentage of Terminal Classic ceramics is lower at Lagunita than at Chactún [15, 46], though this might be due to the limited and unrepresentative amount of ceramics collected at both sites. In sum, the evidence is ambiguous at best.

**Fig 18:**
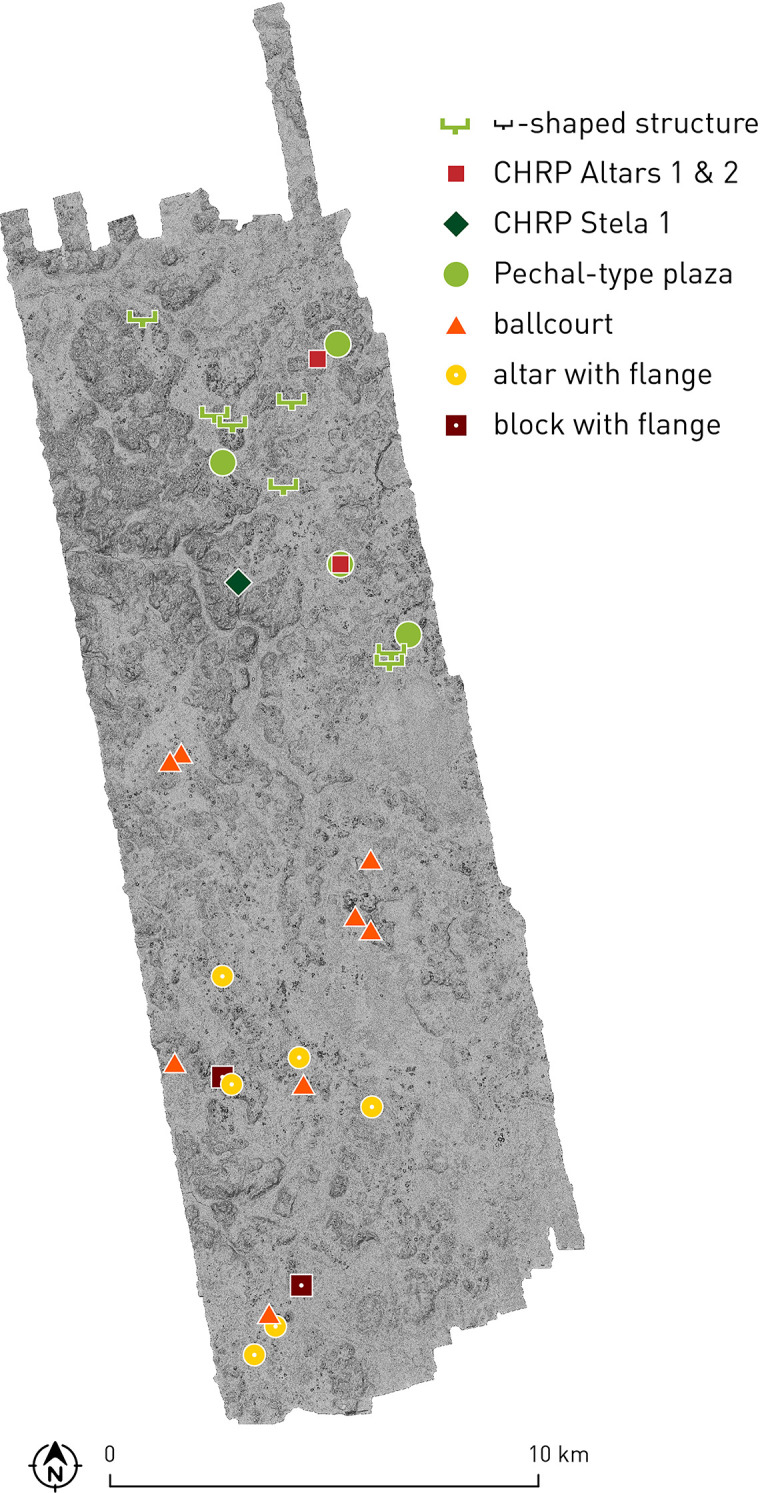
Distribution of certain types of archaeological features, suggesting a political division between Chactún and Pechal.

Whereas the territories that may have been tied to Chactún and Lagunita do not exhibit notable differences in archaeological remains visible on the surface, several types of evidence suggest that the northern part of the CHRP area pertained to a different cultural sphere, likely conditioned by the political domination of Pechal, located about 15 km north-northeast of Chactún (Fig 1). While Pechal has been regularly considered the northernmost Río Bec site [26, 27], its inclusion in the Río Bec sphere has been questioned [31] and, indeed, can hardly be substantiated, not only because of the characteristics of the intermediate CHRP area, but also because Pechal has no temple-like twin towers [23], a hallmark of the Río Bec style and one differentiating it from Chenes architecture [27]. As Fig 18 shows, the Pechal-type plazas, roughly circular and surrounded by almost continuous structures (Fig 6), have only been detected in the northern CHRP area. Also limited to that sector are the buildings with a C-shaped ground plan and an element protruding from the rear side. They might be related to a simpler, T-shaped ground plan, a prototype of which was supposed by Gendrop [27: p. 109, Fig 71a] to be at Pechal. Inversely, the ball courts are relatively common in the southern part of the CHRP area, but absent in the northern part; significantly, there is no ball court at Pechal [23]. Likewise, the altars shaped as a cylinder or cone with a flange at one extreme (reminiscent of a nail head) and similarly rimmed rectangular blocks are only found in the southern part (Fig 14A–14D). Finally, while the so-called stone drums are common throughout the area, they appear in greater numbers in the northeastern part, whereas pyramid temples seem to be slightly less common in the extreme southern part.

Although there is no evidence supporting the existence of strict and enduring boundaries between Maya polities [87], the differential distribution of archaeological remains discussed above suggests that an approximate division line between the communities controlled by Pechal and Chactún during the Late and Terminal Classic roughly coincided with the valley running from the northwestern edge of our area in a southeasterly direction; a relative scarcity of structural remains along this valley, evident in the volume density distribution (Fig 17), is consistent with this proposal. If so, the local lords that commissioned CHRP Stela 1 and Altars 1 and 2 (Figs 11C, 12 and 13) were probably subordinate to Pechal. The fact that these are the only carved monuments found in the area outside Chactún and Lagunita might indicate that only the authorities at Pechal allowed their clients to erect inscribed monuments. While no details of political relations between these centers can be discerned from the data at hand, it is worth noting that no obvious defensive structures have been detected throughout the area. A lack of formal defenses does not necessarily imply the absence of armed conflicts; however, since fortifications do characterize a number of Maya sites, it would seem that the political divisions suggested by our evidence did not entail major hostilities.

## Conclusion

Until a few years ago, a vast area in the central Yucatán lowlands, extending between the relatively well explored Chenes and Río Bec regions in eastern Campeche, Mexico, was an archaeological *terra incognita*, although it lies in the very heartland of the territory once occupied by the Maya. Our surveys in the northern part of the depopulated Calakmul Bioshpere Reserve represented a first attempt to remedy the situation. In 2013 and 2014, we located Chactún, which turned out to be one of the largest sites in the central Maya Lowlands, and Tamchén and Lagunita. Airborne laser scanning (ALS) of the surrounding territory in 2016 revealed a thoroughly modified landscape, practically undisturbed by activities following the Terminal Classic collapse of the Maya culture. We have analyzed the characteristics and distribution of archaeological remains visible on ALS-derived imagery, as well as a large amount of additional information obtained through 2017 and 2018 field surveys, which included test excavations and collection of samples of surface material. The results of this research, in part presented in another publication focused on water management and agricultural strategies [6], have provided important insights into the settlement dynamics in the area, its relations with other parts of the Maya Lowlands, and aspects of social organization and political geography.

The earliest occupation of the area goes back to the early first millennium BCE. In fact, Tamchén and a smaller residential group are the earliest settlements known so far in a broader area in the central lowlands extending within a radius of about 100 km from Tamchén. Interestingly, the Pre-Mamom ceramics found in test pits at both locales is affiliated to the Ek sphere of northern Yucatán [46, 55], having obvious implications for understanding the processes of early colonization of the interior of the Yucatán peninsula. From the early Middle Preclassic to the Late Classic, the CHRP area witnessed a continuous population growth, which was accompanied—and likely conditioned—by increasingly sophisticated techniques of water management and intensive agriculture [6]. Tamchén seems to have been the only major Preclassic center, having strong ties to a broad Petén tradition, but its power waned in the Early Classic, perhaps as a consequence of the processes that started with substantial occupation and construction activity at Lagunita and continued with its florescence and the rise of Chactún in the Late Classic.

Expectedly, the area was closely connected with the nearby Río Bec region, but also, and with differing intensity in different periods, with other parts of the Maya Lowlands. The currently visible architectural remains are largely from the Late Classic, when the population reached its maximum, but do not exhibit a generalized adherence to the specific and in several aspects similar styles that characterize the coeval architecture in the Chenes area to the north and the Río Bec region to the south. While features typical of both styles are represented by various elements of architectural decoration, including the zoomorphic portal of Lagunita [28], both ceramics and other material remains attest to much more variable relations with the surrounding areas. Particularly notable are connections with the Petén, reflected in the omnipresence of pyramidal temples and, most distinctly, in the monumental architecture, urban layout, and the many inscribed monuments of Chactún. If the apogee of Chactún and Lagunita corresponds to the first half of the 8th century, as indicated by the dates recorded on their stelae [3, 34], it may well have been related to the decline of Calakmul, starting with its defeat by Tikal in 695 CE, and possibly involved some immigrations from the Petén [15].

Probably even more surprising is the evidence that the area was exposed, since the earliest times and throughout its evolution, to substantial cultural interaction with, or even migrations from, the northern lowlands. The many annular lime kilns are of the Late Classic type documented in great numbers in northwestern Yucatán peninsula, particularly in the Puuc region, but not elsewhere. The Terminal Classic Xcocom ceramics is clearly intrusive, evidencing the arrival of immigrants from the north and northwest [15]. At Becán this probably occurred in the early 9th century, after a substantial abandonment around 730–750 CE [58]. Only more extensive excavations could reveal whether there was such a gap also in the CHRP area. However, a break in the local tradition attributable to Xcocom intruders is at least suggested by the smashing and resetting of monuments at Chactún and Lagunita, occurring sometime after 751 CE, which is the latest of the dates recorded on these monuments [3, 33]. These events can be interpreted as a sign of the Terminal Classic crisis, reflected in population decrease throughout the area and caused by a combination of stressful circumstances discussed elsewhere [6]. Even during the Postclassic, when the area was almost completely depopulated, its connections with the north persisted, as indicated by the ceramic pieces deposited at some of the ancient monuments, clearly as a part of veneration rituals.

A Terminal Classic intrusion or influence from the north is also evidenced at Kajtún in the Río Bec core zone by the presence of a Fat God sculpture and some syntactic peculiarities in the text on its Stela 6 dated to 795 CE [17, 91]. Other affinities with the north have been noted in iconographic elements on a stela from Pasión del Cristo, located just north of the Río Bec groups [92: p. 143]. It is thus an interesting question whether, or to what extent, influences from the north affected the development of the Río Bec phenomenon. As argued by Taladoire et al. [48], the typical two-tower buildings in the Río Bec zone did not appear before the late 8th century and seem to have emerged in the west before spreading east and north. Could it be that their monumentality and elegance developed from their precursors in the CHRP area? The relatively modest sizes and a rather coarser appearance of the latter are at least suggestive of such a possibility.

Aside from evidencing cultural interaction with other parts of the Maya Lowlands, the Chactún area also exhibits a number of peculiarities, such as quadrangular altars on cylindrical supports, rimmed conical altars, an odd syntax in the text on Lagunita Stela 2, and the glyphs modeled in stucco on some monuments of Chactún [3, 33]. Distinctive features can also be observed in building types and spatial arrangement of residential groups. The currently visible archaeological remains are largely from the Late Classic and, as we have argued, reflect important aspects of social and political organization in that period. The presence of Chactún and Lagunita stands in contrast to the lack of nucleation in the contemporary Río Bec sites. Both centers were evidently major seats of power capable of extracting agricultural surplus from the surrounding communities and recruiting the manpower necessary for some centrally coordinated efforts. Based on spatial distributions of certain archaeological features, we have also attempted to reconstruct the approximate extents of land that were likely controlled by the major polities. On the other hand, various nucleated residential zones with buildings of different types and sizes suggest the existence of a number of communities or neighborhoods composed of several social strata ranging from commoners to elites with differential access to wealth and power. We have interpreted this evidence as reflecting a social organization comparable to House societies or conical clans. Different nobility titles recorded in inscriptions additionally point to a relatively complex sociopolitical hierarchy, although somehow less decentralized than in the Río Bec region.

The results of our study provide a substantial amount of archaeological information on a previously totally unexplored area. Revealing both its specificities and interaction with other parts of the Maya Lowlands, they contribute to the understanding of regional variations in the development of Maya culture. However, only intensive excavations in the area, as well as further surveys in the surrounding regions, will be able to shed light on a number of questions that, due to the limited scope of our research, remain unsolved.
